# Event-related potentials during word mapping to object shape predict toddlers' vocabulary size

**DOI:** 10.3389/fpsyg.2015.00143

**Published:** 2015-02-13

**Authors:** Kristina Borgström, Janne von Koss Torkildsen, Magnus Lindgren

**Affiliations:** ^1^Department of Psychology, Lund UniversityLund, Sweden; ^2^Department of Special Needs Education, University of OsloOslo, Norway

**Keywords:** N400, vocabulary development, shape bias, object recognition, children

## Abstract

What role does attention to different object properties play in early vocabulary development? This longitudinal study using event-related potentials in combination with behavioral measures investigated 20- and 24-month-olds' (*n* = 38; *n* = 34; overlapping *n* = 24) ability to use object shape and object part information in word-object mapping. The N400 component was used to measure semantic priming by images containing shape or detail information. At 20 months, the N400 to words primed by object shape varied in topography and amplitude depending on vocabulary size, and these differences predicted productive vocabulary size at 24 months. At 24 months, when most of the children had vocabularies of several hundred words, the relation between vocabulary size and the N400 effect in a shape context was weaker. Detached object parts did not function as word primes regardless of age or vocabulary size, although the part-objects were identified behaviorally. The behavioral measure, however, also showed relatively poor recognition of the part-objects compared to the shape-objects. These three findings provide new support for the link between shape recognition and early vocabulary development.

## Introduction

What visual cues do children use when learning the meaning of words? The present study used online electrophysiological measures of semantic processing to investigate how toddlers are able to use visual information of overall object shape or separate object features to activate meaning and word representations. Critically, we sought to investigate whether such neural processes are related to vocabulary size and how they change with maturation around the time of the vocabulary spurt.

Toward the end of the second year of life, children learn language at a remarkable pace, on average doubling their rate of word learning between 18 and 24 months compared to the preceding 6 months (Fenson et al., 1994), and show robust ability to fast map novel words to objects (Woodward et al., 1994; Schafer and Plunkett, 1998; Werker et al., 1998; Friedrich and Friederici, 2008; Torkildsen et al., 2008). Fast mapping, a process through which children are able to map a novel word to its referent after very little exposure, seems to be linked to this rapid increase in vocabulary growth. However, it should be noted that initial mappings between novel words and referents observed in laboratory studies of children this age do not necessarily result in long-term learning and the incorporation of the novel item in the lexicon. Rather, young children's learning and retention from fast mapping seems limited to certain circumstances, such as the lack of competition during encoding, and retention improves with age (Horst and Samuelson, 2008; Horst et al., 2010; Friedrich and Friederici, 2011; Bion et al., 2013). There are large individual differences both in vocabulary size and vocabulary growth at specific ages. Although children as a group undergo an acceleration of the rate of word learning around 18 months, often termed the vocabulary spurt, individual children may show a more continuous vocabulary growth, or several separable spurts (Fenson et al., 1994; Dick et al., 2008). The general acceleration of word learning has been attributed to qualitative changes making the learning process more efficient (e.g., Markman, 1990, 1992; Nazzi and Bertoncini, 2003), or alternatively improvements in domain-general learning abilities (e.g., McMurray, 2007; Mayor and Plunkett, 2010).

Regardless of how this early vocabulary growth is best characterized, our understanding of the individual differences in vocabulary development can be advanced by studying how vocabulary size relates to different processes involved in word learning situations. A robust observation in the literature of development is that children (as well as adults) have a shape bias when extending novel nouns to novel object exemplars, and that this bias correlates with vocabulary size (Landau et al., 1988; Poulin-Dubois et al., 1999; Gershkoff-Stowe and Smith, 2004). The shape bias is the tendency to extend newly learned nouns to other objects that are similar in shape, rather than other properties such as size or texture. Interestingly, the emergence of a shape bias coincides with the age and vocabulary size corresponding to the vocabulary spurt, which is typically between 18 and 24 months with a common productive vocabulary threshold at 50–75 words (see Ganger and Brent, 2004 for a review). Basic level nouns tend to dominate the early lexicon, and basic level categories are typically organized by shape (Rosch et al., 1976). Training young children to attend to shape in object labeling situations can lead to improved novel noun generalization as well as an acceleration of vocabulary growth outside the lab (Smith et al., 2002). Consequently, it has been suggested that the shape bias is an attentional mechanism facilitating word learning by focusing the child's attention on similarities in shape, thus leading to a general insight that objects are categorized according to shape. This, in turn, facilitates further word learning by making it easier to generalize from one object to another within the same category (Smith et al., 2002, 2010; Colunga and Smith, 2008).

A number of studies have investigated familiar object recognition based on shape properties, using a task of “shape caricature recognition” (e.g., Smith, 2003; Pereira and Smith, 2009; Yee et al., 2012). This task presents sparse three-dimensional “caricature” versions of objects commonly familiar to young children, and asks children to identify them in a forced-choice task. Smith (2003) found that in children aged 17–25 months vocabulary size was a better predictor than age of performance in this task, both in a version using the object label (“Where is the ice cream?”) and in a non-linguistic play task where children were judged on using the objects in category-appropriate ways (“What can you do with this?”). Critically, a control condition assured that the children were equally good at recognizing typical perceptually rich versions of the objects, regardless of vocabulary size. Since then, several studies have shown that this type of shape caricature object recognition is predictive of vocabulary size (Pereira and Smith, 2009; Yee et al., 2012), and that 2-year-old so-called “late talkers” show deficits in this ability (Jones and Smith, 2005). Eighteen-month-olds that were trained with abstract shape exemplars were better at generalizing a label to a novel exemplar than those trained with typical detailed exemplars (Son et al., 2008).

In an attempt to tease apart different routes to object recognition, Pereira and Smith (2009) compared 18–24 month-olds' ability to recognize familiar objects based on either correct overall shape or correct local details, and found that children with small vocabularies were better at recognition based on local details, even if the overall shape was wrong, while children with large vocabularies performed better when the overall shape was correct, even without the presence of local details. In a different paradigm, Augustine et al. (2011) investigated how 18- to 30-month-olds were able to use information about object part shapes vs. part relations in an object matching task. When given known objects the children were able to use the configural information about the object parts, while novel objects were matched only based on the shapes of individual parts, with no regard for the relations between them. This finding relates to earlier work demonstrating that younger infants (14- and 18-month-olds) were only able to categorize animals and vehicles in the presence of correct parts (legs or wheels), while older infants (22-month-olds) were not dependent on this information (Rakison and Butterworth, 1998). Although some previous studies have demonstrated that already by 10 months children consider correlations among features when categorizing objects (Younger and Cohen, 1983, 1986), these studies have not assessed the referential relation among objects and their labels, which may engage different processes. Taken together, these results have been used to argue that object recognition based on shape is an important developmental step in the process of achieving efficient word learning abilities, and drives the further development of the shape bias. Recognition of shape promotes generalization by being more category-encompassing and less exemplar-specific than specific features or details. Children might thus undergo a development from object recognition based on fragments, local details or parts, to that based on global shape, and this transition supports categorization and word learning (Son et al., 2008; Smith, 2009, 2013; Yee et al., 2012).

Studies on the shape bias and shape caricature recognition have used tasks requiring active responses from the participants. However, our knowledge of early word learning has been greatly enhanced by electrophysiological research on word comprehension and word-object mapping that captures the ongoing learning and comprehension processes in the brain. In this research an electrophysiological component called the N400 has been central. The N400 component was first observed in adults in response to semantically anomalous words in sentence contexts (Kutas and Hillyard, 1980). Subsequent studies have shown that the N400 component is elicited by words and other meaningful stimuli, and reflects semantic processing. It is modulated by semantic priming and/or ease of semantic integration into context (for recent reviews, see Lau et al., 2008; Kutas and Federmeier, 2011). During the last two decades, a number of studies have investigated the maturation of the N400 in infants and children. It appears that the component may be elicited from approximately 6–9 months of age in picture-word congruity paradigms if the experimental circumstances are favorable (Friedrich and Friederici, 2011; Junge et al., 2012). However, in more demanding versions of this paradigm, the component may not be observed even at 12 months (Friedrich and Friederici, 2005b) and in late talkers and children with dyslexia in the family, the N400 has been found to be absent well into the second year (Friedrich and Friederici, 2006; Torkildsen et al., 2007). Several studies of older children and adolescents suggest that the N400 has a long developmental time course and may not be fully mature before around 19 years of age (Holcomb et al., 1992; Juottonen et al., 1996; Atchley et al., 2006).

As the N400 component has become better documented in children, it has become increasingly clear that it may serve as a useful tool in the study of early lexical-semantic development. Importantly, a number of studies have found links between the N400 and vocabulary size (Mills et al., 2005; Torkildsen et al., 2008, 2009; Friedrich and Friederici, 2010; Junge et al., 2012; Rämä et al., 2013). Early N400 responses have also been found to predict later language abilities (Friedrich and Friederici, 2006). The nature of the relation between the N400 and vocabulary knowledge is not entirely clear. However, it is unlikely that the missing N400 in toddlers with small vocabularies is due to a lack of the N400 mechanism itself, for instance due to an immaturity of the underlying brain structures. For example, 12-month-olds who did not show an N400 effect to picture-word pairs, did show an N400 effect to inappropriate picture-natural sound pairs (e.g., a barking or ringing) (Babocsai et al., 2007, in Friedrich, 2011). Also, the recent studies demonstrating N400 effects to word stimuli in infants (6- and 9-month-olds) show that the specific demands and conditions during experiments are critical to whether an N400 effect is observed (Friedrich and Friederici, 2011; Junge et al., 2012).

The aim of the present study was to test the relation between vocabulary development and the ability to map words onto objects with overall shape or specific parts as cues. We contrasted the neural processes of word comprehension in contexts where images contained only overall object shape information (silhouettes) and contexts where images contained only fragmented details of objects. We had two hypotheses motivated by previous research: (1) that object shape recognition is positively correlated with vocabulary size, and is a better predictor of vocabulary than recognition of regular pictures, (2) that children progress from object recognition based on fragments of objects to that based on shape. In addition to a behavioral task of explicit object identification, similar to that used in previous studies, we used electrophysiological measures of semantic processing that have the advantage of providing graded responses with a high temporal resolution and thus reveal more subtle differences between children at different stages of language development. Differences between children in semantic processing may be seen as amplitude differences in ERP effects, which indicates differential strength of underlying cognitive processes, or different topographies, which can be a sign of different levels of maturation of the brain regions involved or the engagement of different processes and brain regions. This is the first study to investigate shape recognition and vocabulary development using ERP measures. In order to capture the dynamics of development around the vocabulary spurt, the experimental design was longitudinal where children were presented with the same task at 20 and 24 months of age. In population estimates of productive vocabulary in Swedish children, the 50th percentile at 20 months is at 74 words, which would mean that approximately half of the children in this sample would be expected to be at a pre-vocabulary spurt stage (using the common threshold of 75 words). At 24 months, more than 80% of Swedish children are estimated to have vocabularies above 75 words (Eriksson and Berglund, 2002). The experimental task was based on the paradigm developed by Torkildsen et al. (2008, 2009), which was designed to study the fast mapping process in toddlers. An initial learning phase of the experiment established associations between images of common objects and their correct labels, and in a subsequent test phase the pictures were presented with incongruous words in order to elicit an N400 incongruity effect. Additionally, modified versions of the original images, which contained reduced visual information, were also presented with congruous and incongruous word pairs. We expected children with larger vocabularies, especially in comparison to children with vocabulary sizes below 75 words, to show a stronger N400 incongruity effect in the silhouette condition specifically. If children progress from object recognition based on parts or fragments early in development (age and vocabulary) to that based on shape, we should see an N400 effect in the detail condition regardless of age and vocabulary, and perhaps a stronger effect in children with smaller vocabularies. Based on previous research we expected children at both ages to display an N400 effect in the regular picture condition, independent of vocabulary size (Friedrich and Friederici, 2004, 2005a, 2006, 2008, 2010; Torkildsen et al., 2006, 2007, 2008).

**Figure 1:**
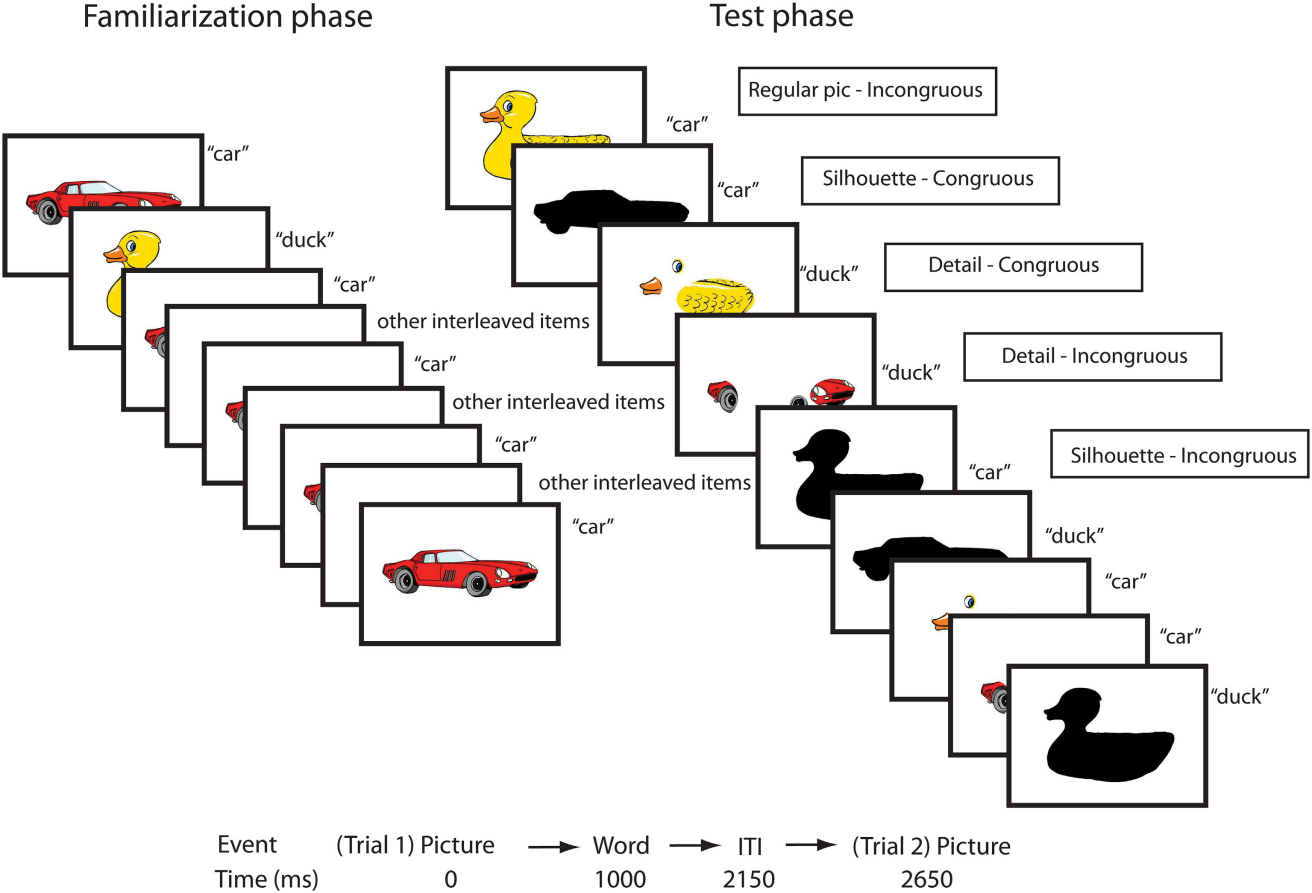
**Illustration of the block design in the ERP experiment, with the different conditions in each phase**. There was always at least one interleaved item between item repetitions, and each picture-word pair (three real words and their referents in each block) was presented five times in the familiarization phase.

A difference between the present and previous studies is that the object parts condition employed images with only a few salient details, isolated from each other (see Figure 1), in order to test object recognition from fragments rather than configural information about object parts. Previous studies have used objects with incorrect configuration of parts (Pereira and Smith, 2009; Augustine et al., 2011; Yee et al., 2012). The object parts have been connected in a unified object, but results from these studies have been taken as evidence for an early object recognition described as based on “fragments” (Smith, 2009), although a fragment is typically understood as “a small part broken off or detached” (American Heritage Dictionary, 2004). The present study's stimulus material was an attempt to test children's interpretation of object fragments specifically.

## Materials and methods

### Participants

#### 20 months

The total sample of participants consisted of 77 children (36 boys) at 20 months of age (±3 weeks), of which 56 were included in either the electrophysiological or behavioral analysis. Only typically developing monolingual Swedish speaking children born at full term (>36 gestational weeks) were included. Of these, 45 children completed the behavioral experiment (17 boys), and reliable electrophysiological data was obtained from 38 children (17 boys). The other 39 children were excluded from EEG analysis due to fussiness, technical problems, or too few artifact-free trials in one or more of the analyzed conditions. Attrition in the behavioral experiment was mainly due to inability to consistently perform the required response (pointing to the labeled object). Developmental data from questionnaires was obtained for 74 children. The children were recruited mainly through child health care centers in and around Lund, Sweden, and an information campaign sent by mail to all children in certain areas close to Lund that would fall within the appropriate age range during the study period. The project was granted ethical approval by the Regional Ethical Review Board, according to the decision DNR-2009-383.

#### 24 months

The follow-up experimental session included 54 children (24 months ±3 weeks), of which three were excluded from all analyses. The behavioral experiment was completed by 48 children (21 boys) and reliable electrophysiological data was obtained from 34 children (15 boys). Twenty children were excluded from the ERP analysis due to reasons stated above. Longitudinal ERP data was obtained from 24 participants (i.e., the subset of participants that overlapped at 20 and 24 months). Of the additional 10 participants in the ERP sample, eight had participated at 20 months without fulfilling the inclusion criteria, and two children were allowed to enter the study only for the 24 month session in order to increase the sample.

### Materials

Three parent questionnaires were used to assess the children's general level of development. The Swedish Early Communicative Development Inventory (SECDI) (Eriksson and Berglund, 2002), a Swedish adaptation of MacArthur-Bates Communicative Development Inventories (Fenson et al., 1993), was used (the Words and Sentences version which assesses language production skills, designed for children over 16 months). Additionally, we used the 20 and 24 months versions of a Swedish adaptation of the Norwegian Ages and Stages Questionnaires (ASQ) (Squires et al., 1999; Janson and Smith, 2003), which assesses the infant's level of development in various areas including language and motor development.

The stimulus material was different in the 20 and 24 month sessions. Please see the Supplementary Materials 2, 3 for complete lists of the items used. The auditory stimulus material for the ERP experiment at both time points consisted of 30 common count nouns (15 artifact labels and 15 animal labels). Parents reported that their children comprehended on average 21 of these words at 20 months (range = 10–28), and 26 words at 24 months (range = 6–30). All words were recorded in an anechoic chamber by a female voice, speaking in an infant-directed manner. For the behavioral task, 18 common nouns, artifacts and animals, were included at 20 months, and 14 nouns at 24 months (the task was shortened in order to decrease attrition). The visual stimulus material consisted of cartoon images of the objects corresponding to the chosen nouns selected from the web-based collection Clipart (Vital Imagery Ltd.)^1^. Two modified versions of the pictures were created, one displaying only a few isolated parts (showing between 30 and 40% of the original picture), and one displaying a black, filled silhouette of the object (see Figure 1). A pilot-test where a sample of children in the targeted age group were asked to point to a named object among several pictures, showed that the modified pictures were generally recognizable.

Cartoon-images of the objects were used rather than realistic photographs for several reasons. First, we wished to use similar stimuli as many of the previous experiments involving picture-word matching (e.g., Friedrich and Friederici, 2004, 2008, 2011; Torkildsen et al., 2008, 2009), and the drawings also seemed to better resemble the type of toy stimuli used in behavioral experiments on shape recognition (Pereira and Smith, 2009; Augustine et al., 2011). Moreover, many children's books have illustrations rather than photographs, which makes it likely that toddlers would be familiar with the procedure of labeling and pointing to such pictures.

### Procedure

#### EEG experiment

The child sat on the parent's lap, with a white cardboard screen placed around them in order to block out distractions. The light was dimmed so that the stimuli would become more salient, and to help the child stay focused on the screen. EEG data was recorded with infant versions of the 128 channels HydroCel Geodesic Sensor Nets (Electrical Geodesics, Inc.) connected to a Net Amps 300, with a sampling rate of 250 samples/s, referenced to the vertex. Impedances were kept below 50 kΩ, according to recommendations from the manufacturer. The stimulus material consisted of a slideshow of pictures presented on a 17 inch computer screen (34 × 27 cm) positioned approximately 35 cm from the child, and auditory presentation of words from a speaker placed in front of the child. There were 10 blocks, allowing for a short break in between blocks if necessary. During breaks, a short animated film clip could be shown if the child seemed tired or unfocused, in order to energize and reorient the child to the screen. The experiment included both real words and their referents, and pseudowords paired with fantasy objects. Only data from the real word conditions are presented in this paper. The pseudoword conditions were included to study the novel word learning process, and this data will be presented in another paper. Each block consisted of three real words and three pseudowords and their picture referents. Each picture-word pair was presented five times in a pseudorandomized order, specified so that each block began with a real word, there was always at least one interleaved item in between item repetitions, and there were at most two real word trials or pseudoword trials in a row. Two different trial lists were used, containing the same stimuli but different orders of presentation. Pictures were presented for 2150 ms, with a word onset of 1000 ms after each picture onset, in order to create a priming context for the word. In between each trial a white screen was displayed for 500 ms. The last part of each block was a test phase, where each picture was presented together with incongruous words from the same block, and the two modified versions of each picture were presented in a congruous condition (with the correct label) and an incongruous condition. A video camera placed by the screen recorded the child's behavior, enabling the exclusion of trials where the child was inattentive.

#### Behavioral experiment

The ERP experiment was performed before the behavioral experiment. The child was seated on a parent's lap at a table opposite the experimenter. The picture stimuli were arranged 3 by 3 on a size A4 white cardboard paper, and only pictures from the same category (detail or silhouette) were presented at the same time. The experimenter presented the cardboard papers one by one. For each presentation, the child was asked to point out the correct picture of the object named (“Where is the dog?” or “Can you find the dog?”), among the two distractors. The choice of distractors, and the arrangement of the three pictures, was randomized, as well as the order of the items. We created two versions of the randomization and allocated half of the participants to each list. The child only saw one version of each object (silhouette or detail). As a control condition, there were also displays of each object in its complete version. The objects that the child was not able to identify correctly were presented at the end of the task in their complete versions, and the child was once again asked to point out the correct picture among two distractors. This test served as a baseline to assess which words the child really knew and was able to point out. The session was video-recorded to enable subsequent analysis and coding.

### Analysis

#### EEG analysis

The video from each experimental session was viewed time-locked to the session's EEG data, and sections of inattentiveness (e.g., the child looking away, crying or yawning) were rejected before further analysis. The EEG data was filtered using a finite impulse response filter with a bandpass of 0.3–30 Hz. Epochs were created time-locked to word onset, lasting for 1250 ms with a 100 ms pre-stimulus baseline. Large artifacts and bad channels were detected automatically (defined as max-min voltage changes >200 μV) in Net Station 4.5 (Electrical Geodesics Inc.) and trials with more than 15 bad channels were rejected. Individual trials were then checked visually to confirm the bad channel detection and to adjust it so that it did not include artifacts caused by eye blinks and eye movements (these were left in the data for later correction). Remaining bad channels were replaced using spherical spline interpolation. Data was re-referenced to the average of all electrodes (excluding vertical and horizontal eye electrodes and the nasion electrode). The choice of average reference has been argued to be the least biased when including a large number of electrodes (e.g., Picton et al., 2000). An individual components analysis in EEGLAB (Delorme and Makeig, 2004) was performed to identify and remove eye blink and eye movement components, and remaining analyses were performed in ERPLAB (Lopez-Calderon and Luck, 2014). Only data from subjects who retained at least 10 artifact-free trials per condition were included in the grand average and the statistical analyses. The mean number of accepted trials per condition at 20 months was between 15 and 17 for all analyzed conditions, and between 14 and 16 at 24 months.

***Statistical analysis***. Nine regions of interest (ROI), each including six electrodes, were selected that covered left, midline, and right sections of frontal, central, and parietal regions. These regions were selected due to the N400 component's typical topography over centro-parietal regions, and occasionally in young children frontal regions (Kutas and Federmeier, 2011). The average amplitude for the six electrodes was calculated to obtain one measure for each region for use in statistical analyses. See the Supplementary Material 1 for channel layout and the electrodes included in each ROI. Only ERP data from the test phase and the final presentation of the learning phase (incongruous vs. congruous presentations) are reported in this paper. For regular pictures, the incongruous presentation was compared to the fifth and final congruous word presentation, since children at this point reasonably would have reached similar levels of familiarization with the stimuli. The time period of 500–900 ms was chosen for analysis, based on the typical timing of N400-effects in this age group (Friedrich and Friederici, 2004, 2010; Torkildsen et al., 2008). This time period corresponded well with visual inspections of the waveforms. After performing an omnibus ANOVA including all conditions and electrode regions, analyses were focused on testing central and parietal sites specifically, since these regions showed the largest effect due to incongruity.

First, a five-way repeated measures ANOVA was performed, with Picture Type (3 levels), Congruity (2 levels), Region (3 levels), and Laterality (3 levels) as within-subject factors, and vocabulary group (2 levels) as between-subject factors. Follow-up three-way ANOVAs were performed in each of the three conditions (regular, detail and silhouette pictures), at central and parietal regions separately, to test for the effect of congruity (2 levels: congruous, incongruous), and its interaction with laterality (3 levels: left, midline, right) as within-subject factors, and productive vocabulary group (2 levels: high, low) as between-subject factor. In cases of significant interactions, follow-up ANOVAs on each group or each lateral electrode site were performed separately. Only significant effects and certain effects approaching significance (*p* < 0.100) that include the congruity factor are reported. An alpha-level of 0.05 was used for all statistical tests. The Huyn-Feldt Bound correction was used when the assumption of sphericity was violated, and in these cases unadjusted degrees of freedom and adjusted *p*-values are reported.

#### Vocabulary measures

To investigate the relation between experimental effects and productive vocabulary, participants were divided into two groups based on their SECDI total productive vocabulary score according to a median split. This was entered as a group factor in the statistical analyses. Critical experimental measures that were found to interact with vocabulary group were then followed-up in simple regression analyses to explore relations with the continuous measure of vocabulary size. We also explored using only the number of object count nouns as an alternative vocabulary measure, as this has been shown to be particularly relevant to the development of a shape bias (e.g., Pereira and Smith, 2009; Perry et al., 2010; Perry and Samuelson, 2011). However, this measure was found to correlate very strongly with the total vocabulary measure, and substituting this measure for the total vocabulary measure in the analyses did not alter any of the results.

#### Behavioral experiment

Children's pointing responses were scored as correct or incorrect. A response was marked as correct if the correct picture was the first that the child pointed to. If the child pointed to another picture first, and then the correct picture, the response was marked as correct only if the child also said the correct word when pointing to the picture, or named the other picture correctly and then when asked a second time pointed to the correct picture. All other responses, or the failure to respond, were marked as incorrect. Finally, each child received one score for the detail pictures and one for the silhouettes, where incorrectly identified objects that the child was not able to identify in the regular picture version were excluded.

## Results

### ERP results

Results from the omnibus ANOVA for mean amplitudes between 500 and 900 ms are presented in Table 1. There were significant main effects of congruity (greater negativity in incongruous condition) at both ages, as well as interactions between congruity and region and near significant interactions with vocabulary group. At 20 months there was an interaction between congruity and picture type. Therefore, we continued to analyze each condition separately, and central and parietal regions specifically, as the interactions with region showed largest differences due to incongruity over these sites.

**Table 1:** **Results from omnibus ANOVA**.

	**20 months**					**24 months**				
	***F***	***p***	**η^**2**^_***p***_**	***df***	**Error df**	***F***	***p***	**η^**2**^_***p***_**	***df***	**Error df**
Congruity	7.043	0.012	0.164	1	36	10.71	0.003	0.251	1	32
PicType × Congruity	4.62	0.013	0.114	2	72		n.s.			
PicType × Congruity × Region	3.55	0.011	0.090	6	216		n.s.			
Congruity × Region × Laterality × PVGroup	2.039	0.093	0.054	6	216		n.s.			
Congruity × Region		n.s.				4.02	0.028	0.112	3	96
Congruity × Region × PVGroup		n.s.				2.81	0.076	0.081	3	96

#### Incongruity effects to regular pictures

**Figure 2:**
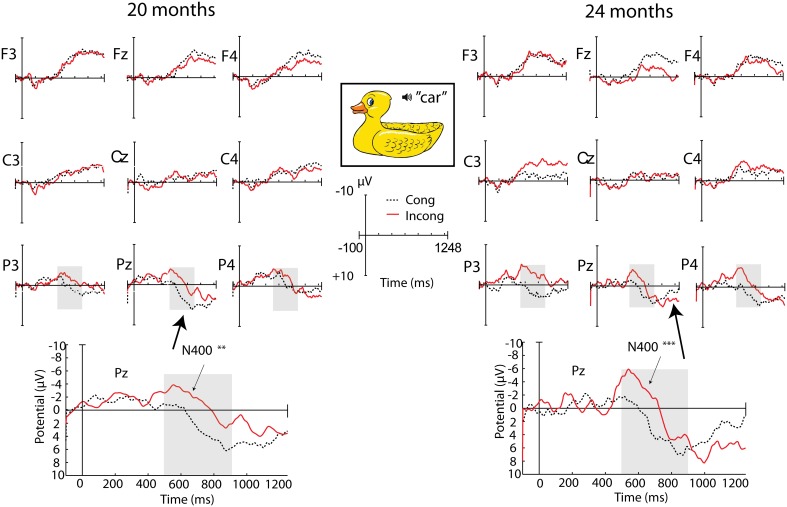
**ERPs to incongruous vs. congruous words presented with regular pictures**. Regions and time window with significant effect of congruity are shaded. ^**^p < 0.03; ^***^p < 0.01.

Incongruous words elicited a large negativity over posterior sites lasting from approximately 400–900 ms, with a peak around 600 ms (see Figure 2). At both 20 and 24 months there was a main effect of congruity at parietal electrodes: 20 months, *F*_(1, 36)_ = 7.50, *p* = 0.010, η^2^_*p*_ = 0.172; 24 months, *F*_(1, 32)_ = 15.50, *p* < 0.001, η^2^_*p*_ = 0.320. There were no significant interactions with vocabulary group at either age.

#### Incongruity effects to silhouette pictures

Incongruous words presented with silhouette pictures elicited a large negativity compared to congruous words, distributed across posterior and central regions. The negative wave lasted from approximately 400 ms throughout the epoch, with a pronounced peak around 600 ms at parietal sites, but with a longer-lasting negativity at central sites. A summary of the results from the statistical analyses at both ages are reported in Table 2.

**Table 2:** **Results from repeated measures ANOVA in the silhouette condition**.

**Full sample**	**20 months**					**24 months**				
	***F***	***p***	**η^**2**^_***p***_**	***df***	**Error df**	***F***	***p***	**η^**2**^_***p***_**	***df***	**Error df**
**PARIETAL**										
Congruity	10.20	0.003	0.221	1	36	7.54	0.010	0.191	1	32
**CENTRAL**										
Congruity	7.81	0.008	0.178	1	36	4.45	0.043	0.122	1	16
Congruity × Laterality	5.48	0.008	0.132	2	72		n.s.			
Congruity × PVGroup	3.30	0.077	0.084	1	36	4.40	0.044	0.121	1	16
**LOW VOCABULARY GROUP**										
Congruity (Central)	0.81	0.380	0.43	1	18	<0.001	0.995	<0.001	1	16
**HIGH VOCABULARY GROUP**										
Congruity (Central)	7.53	0.013	0.295	1	18	13.63	0.002	0.460	1	16

***20 months***. There was a main effect of congruity over parietal regions, with no significant interactions. At central sites, there was a main effect of congruity, an interaction between congruity and laterality, and a near-significant interaction with vocabulary group. Follow-up analyses showed that the effect of congruity was only significant at midline, *F*_(1, 36)_ = 10.60, *p* = 0.002, η^2^_*p*_ = 0.224 and right central electrodes, *F*_(1, 36)_ = 9.39, *p* = 0.004, η^2^_*p*_ = 0.207, and at midline electrodes there was also a significant interaction with vocabulary group, *F*_(1, 36)_ = 4.12, *p* = 0.050, η^2^_*p*_ = 0.228. Separate group analyses revealed that only the high vocabulary group had a significant effect of congruity at this region, *F*_(1, 18)_ = 10.45, *p* = 0.005, η^2^_*p*_ = 0.367 [LV group: *F*_(1, 18)_ = 1.14, *p* = 0.301, η^2^_*p*_ = 0.059]. This group difference held true for the entire central region as well (see Table 2).

**Figure 3:**
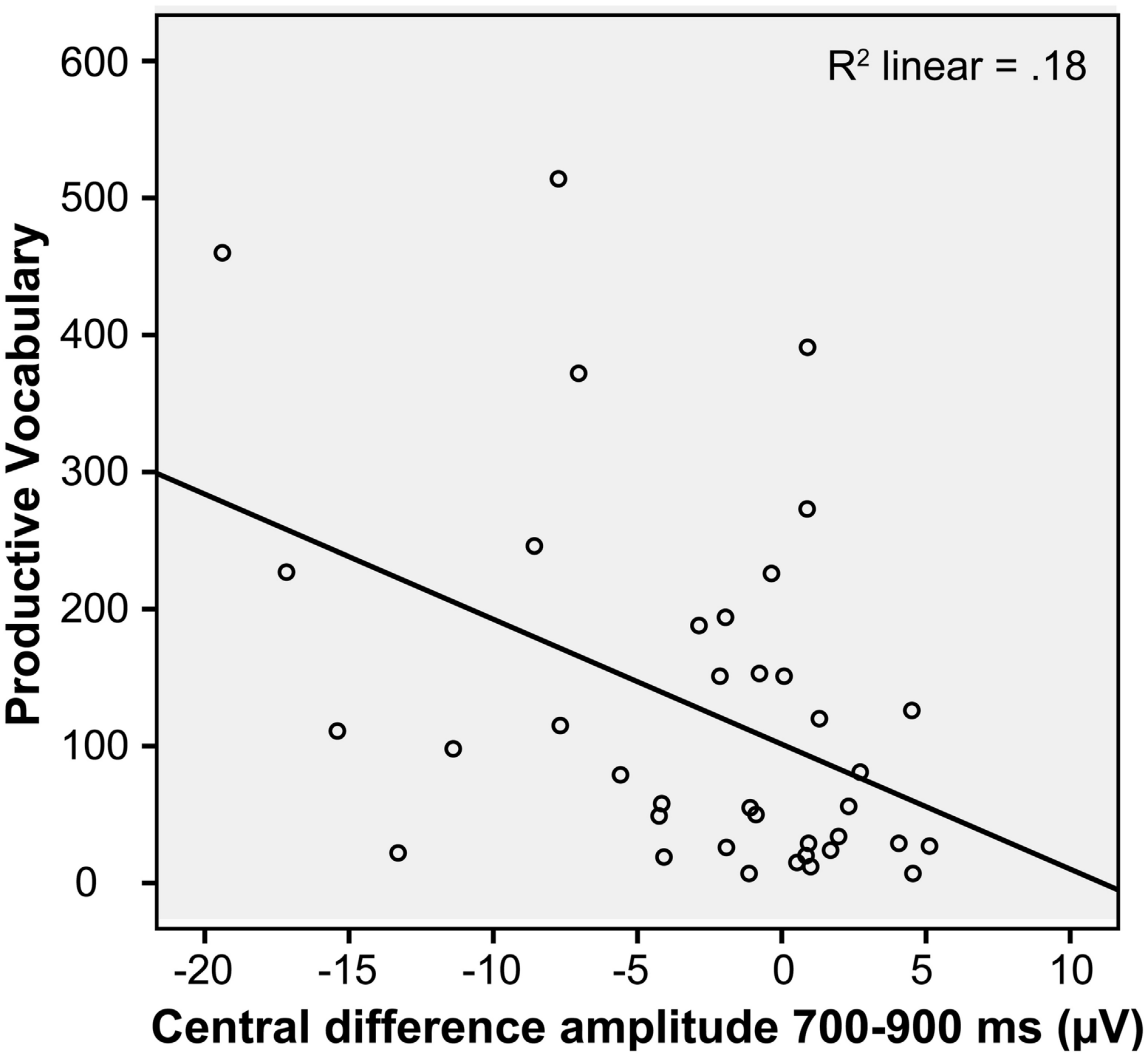
**Relation between productive vocabulary and the difference in amplitude between congruous and incongruous words in the silhouette condition (in the 700 and 900 ms time window over central electrodes), at 20 months**. Note that a negative difference score corresponds to a greater negativity to the incongruous presentation, while a positive difference score corresponds to the opposite pattern of response.

As a follow-up analysis to investigate the relation between the silhouette central incongruity effect and vocabulary, we performed a regression analysis to see if the mean difference amplitude (incongruous minus congruous) over central sites between 500 and 900 ms correlated with the continuous measure of productive vocabulary. There was a significant relation, *r* = −0.375, *p* = 0.020. Children with a larger negativity to the incongruous word tended to have larger vocabularies, and the effect was even stronger in a more narrow time window, 700–900 ms, *r* = −0.43, *p* = 0.008 (see Figure 3).

**Figure 4:**
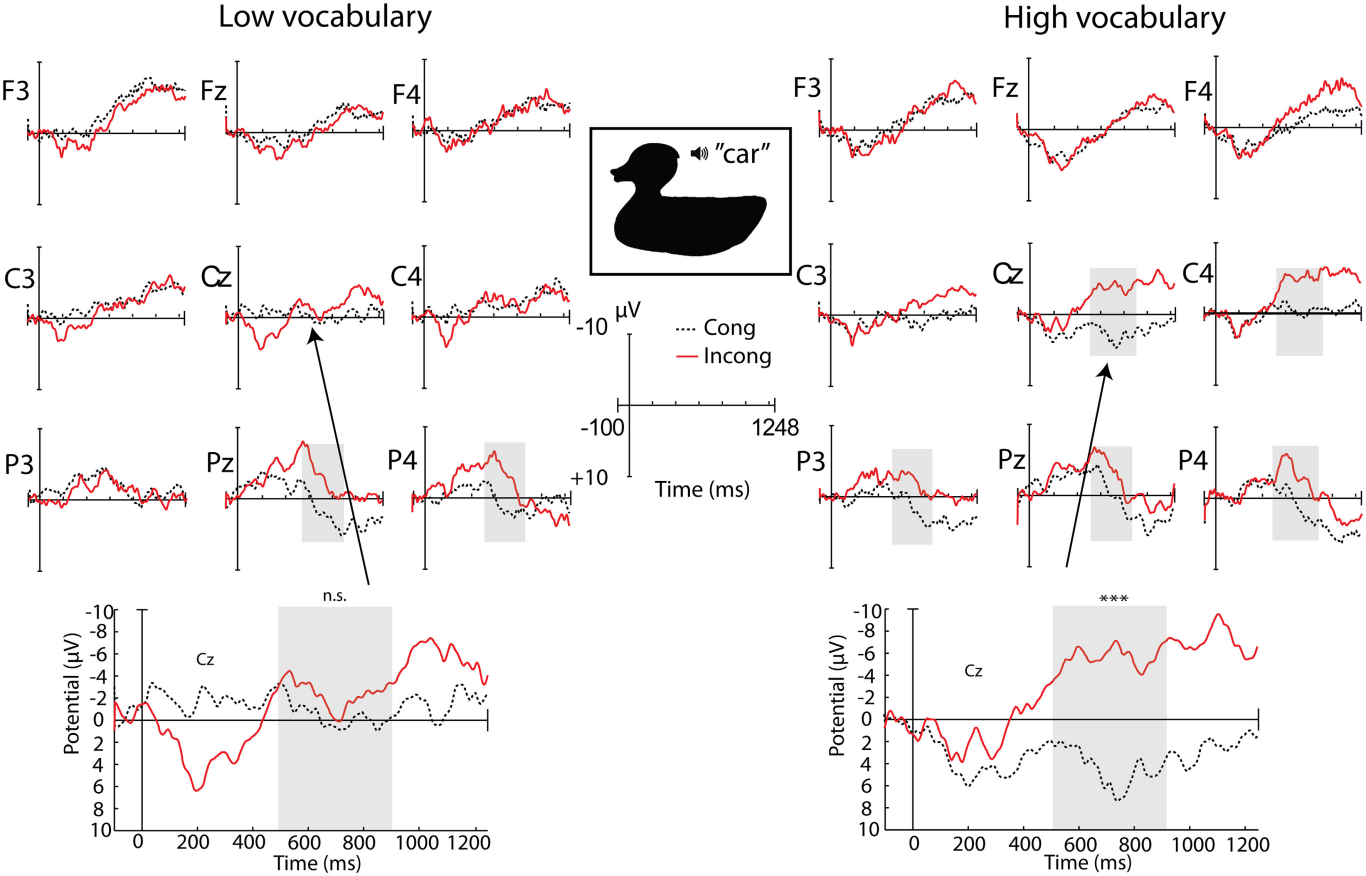
**ERP topography for the congruous and incongruous silhouette conditions at 20 months, displayed for the two vocabulary groups separately**. The time window with a significant effect of congruity in separate group analyses is shaded in gray (^***^p < 0.01).

In sum, children displayed a parietal N400 component to incongruities in the silhouette condition regardless of vocabulary size. However, the size of the incongruity effect over central electrode sites correlated with vocabulary size, where a larger effect was associated with a larger vocabulary size. At the group level, children with smaller vocabularies only had a parietal N400 effect, while children with larger vocabularies showed a parietal N400 effect as well as a central negativity with a relatively right lateralized distribution (see Figure 4).

**Figure 5:**
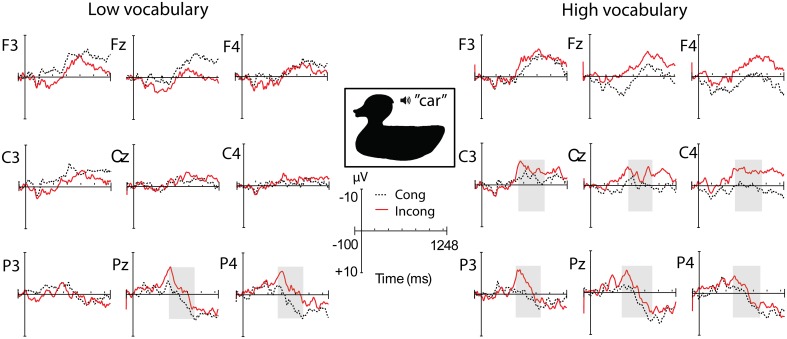
**ERP topography for the congruous and incongruous silhouette conditions at 24 months, displayed for the two vocabulary groups separately**. Time windows with significant effects in separate group analyses are shaded in gray.

***24 months***. Over parietal regions, there was a main effect of congruity, with no significant interactions (see Table 2 and Figure 5). At central sites, there was also a main effect of congruity, as well as a significant interaction with vocabulary group. As was the case at 20 months, only the HV group had a significant effect of congruity over central sites.

We tested for a correlation between the central amplitude difference between 500 and 900 ms and productive vocabulary at 24 months, but this relation was not significant, *r* = −0.258, *p* = 0.140. There was no significant correlation in the more narrow time window either, 700–900 ms (*r* = −0.217, *p* = 0.218), in which the relation was strongest at 20 months.

#### Incongruity effects to detail pictures

**Figure 6:**
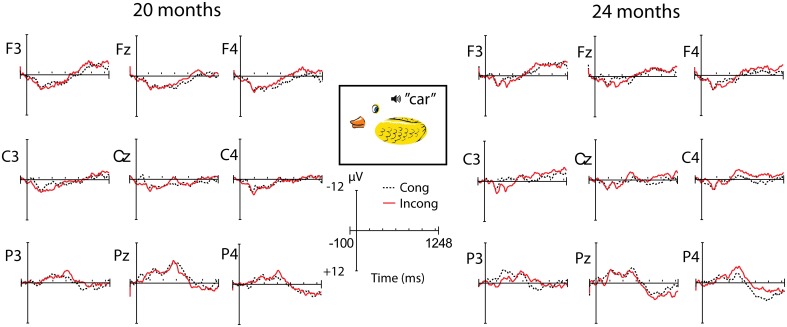
**ERPs to congruous and incongruous words presented with detail pictures, at 20 and 24 months**.

There was no clear difference in ERP waveforms to congruous and incongruous words presented together with detail pictures, at either 20 or 24 months (see Figure 6), and in the tested 500–900 ms time window there were no significant effects of congruity at either age or region (all *p*-values above 0.19), and no interaction with vocabulary group.

### Behavioral results

Results from the behavioral measures, based on questionnaire responses and the behavioral experiment, are reported in Table 3. Scores are reported for the entire sample at each age and for the groups of participants that were included in the ERP analyses separately. As can be seen in Table 3, there was no difference in vocabulary size between participants who contributed to the ERP data and those who did not.

**Table 3:** **Behavioral measures—descriptive statistics**.

**Scale**	**Measure**	**Full sample**				**ERP sample**			
		***n***	**Median**	**Mean (*SD*)**	**Range**	***n***	**Median**	**Mean (*SD*)**	**Range**
**20 MONTHS**									
ASQ	Communication	74	45	41.89 (13.96)	5–60	38	45	41.18 (14.54)	5–60
ASQ	Gross motor	74	60	54.32 (7.95)	25–60	38	55	53.42 (8.15)	30–60
ASQ	Fine motor	74	50	49.93 (9.12)	30–60	38	55	50.92 (9.36)	30–60
ASQ	Problem solving	74	45	43.45 (7.76)	25–60	38	45	44.47 (7.42)	30–60
ASQ	Personal/Social	74	50	49.46 (7.66)	30–60	38	50	49.47 (7.69)	35–60
SECDI	Prod. vocabulary	74	66	110 (112)	1–514	38	80	127 (131)	7–514
Behav. Experiment	Silhouette	45	0.89	0.86 (0.14)	0.50–1.00	27	1.00	0.90 (0.14)	0.56–1.00
Behav. Experiment	Detail	45	0.67	0.69 (0.17)	0.25–1.00	27	0.71	0.71 (0.17)	0.25–1.00
LV group	Prod. Vocabulary					19	27	33 (20)	7–79
HV group	Prod. Vocabulary					19	188	221 (127)	81–514
**24 MONTHS**									
ASQ	Communication	54	55	52.13 (11.06)	20–60	34	57	53.09 (10.52)	20–60
ASQ	Gross motor	54	60	55.65 (5.99)	35–60	34	60	55.29 (6.74)	35–60
ASQ	Fine motor	53	55	53.02 (6.23)	35–60	33	55	53.18 (6.10)	35–60
ASQ	Problem solving	53	50	48.40 (9.29)	25–60	33	50	50.30 (8.10)	30–60
ASQ	Personal/Social	54	50	50.37 (6.86)	35–60	34	50	50.15 (7.33)	35–60
SECDI	Prod. vocabulary	54	315	291 (166)	6–605	34	318	296 (163)	15–605
Behav. Experiment	Silhouette	48	1.00	0.92 (0.11)	0.57–1.00	31	1.00	0.93 (0.11)	0.57–1.00
Behav. Experiment	Detail	48	0.86	0.85 (0.16)	0.50–1.00	31	1.00	0.85 (0.15)	0.57–1.00
LV group	Prod. vocabulary					17	196	163 (101)	15–317
HV group	Prod. vocabulary					17	391	428 (84)	318–605

#### Behavioral experiment

***20 months***. An independent-samples *t*-test comparing the mean productive vocabulary scores between participants who completed the behavioral experiment and those who did not, showed that there was a significant difference between the groups [*t*_(72)_ = 4.26, *p* < 0.001, *d* = 1.04]. The group that completed the behavioral experiment had a mean score of 148 words (*SD* = 124) compared to the rest of the sample: *M* = 55 (*SD* = 62).

The effect of condition on object identification was tested using a repeated measures ANOVA with silhouette and detail as the two levels of condition and productive vocabulary group (based on a new median-split on the sample that completed the behavioral experiment) as a between-subjects factor. A one-sample *t*-test was performed to confirm that children performed above chance. Children were better able to identify objects from silhouette (Sil) pictures than detail (Det) pictures, *F*_(1, 43)_ = 31.41, *p* < 0.001, η^2^_*p*_ = 0.422, but both conditions were identified significantly better than the 33% chance level [Sil: *t*_(44)_ = 24.61, *p* < 0.001, *d* = 3.79; Det: *t*_(44)_ = 14.23, *p* < 0.001, *d* = 2.12]. There was no significant interaction between condition and vocabulary group. We had predicted a correlation between vocabulary and silhouette recognition particularly, but neither condition correlated significantly with vocabulary. However, recognition based on silhouettes, but not recognition based on details, correlated significantly with our other language measure, ASQ Communication (*r* = 0.32, *p* = 0.034).

Given that the behavioral experiment and the ERP experiment were designed to test the same ability to recognize an object and retrieve its verbal label, although provide different measures of the processes involved, we expected to see a relation between behavioral performance in the silhouette condition and N400 difference amplitude between congruous and incongruous presentations. As predicted, there was a significant correlation between the behavioral silhouette score and the mean parietal amplitude difference (incongruous minus congruous) between 500 and 900 ms (*r* = −0.40, *p* = 0.038). Thus, children who were better able to point to the correct silhouette image were more likely to have large differences in N400 amplitude over parietal regions.

***24 months***. The effect of condition on object identification was tested using a repeated measures ANOVA with silhouette and detail as the two levels of condition and productive vocabulary group as a between-subjects factor. A one-sample *t*-test was performed to confirm that children performed above chance. Children were better able to identify objects from silhouette pictures than detail pictures, *F*_(1, 46)_ = 9.24, *p* = 0.004, η^2^_*p*_ = 0.167, but both conditions were identified significantly better than the 33% chance level [Sil: *t*_(47)_ = 36.73, *p* < 0.001, *d* = 5.36; Det: *t*_(47)_ = 22.99, *p* < 0.001, *d* = 3.25]. There was no significant interaction between condition and productive vocabulary group. Performance in both the silhouette and detail conditions correlated positively with productive vocabulary (Sil: *r* = 0.408, *p* = 0.004; Det: *r* = 0.340, *p* = 0.017). Only the silhouette condition correlated positively with the ASQ Communication scale, *r* = 0.39, *p* = 0.006. No correlation was found between performance in the silhouette condition and ERP difference amplitude as an effect of incongruity in silhouette contexts.

### Longitudinal results

**Figure 7:**
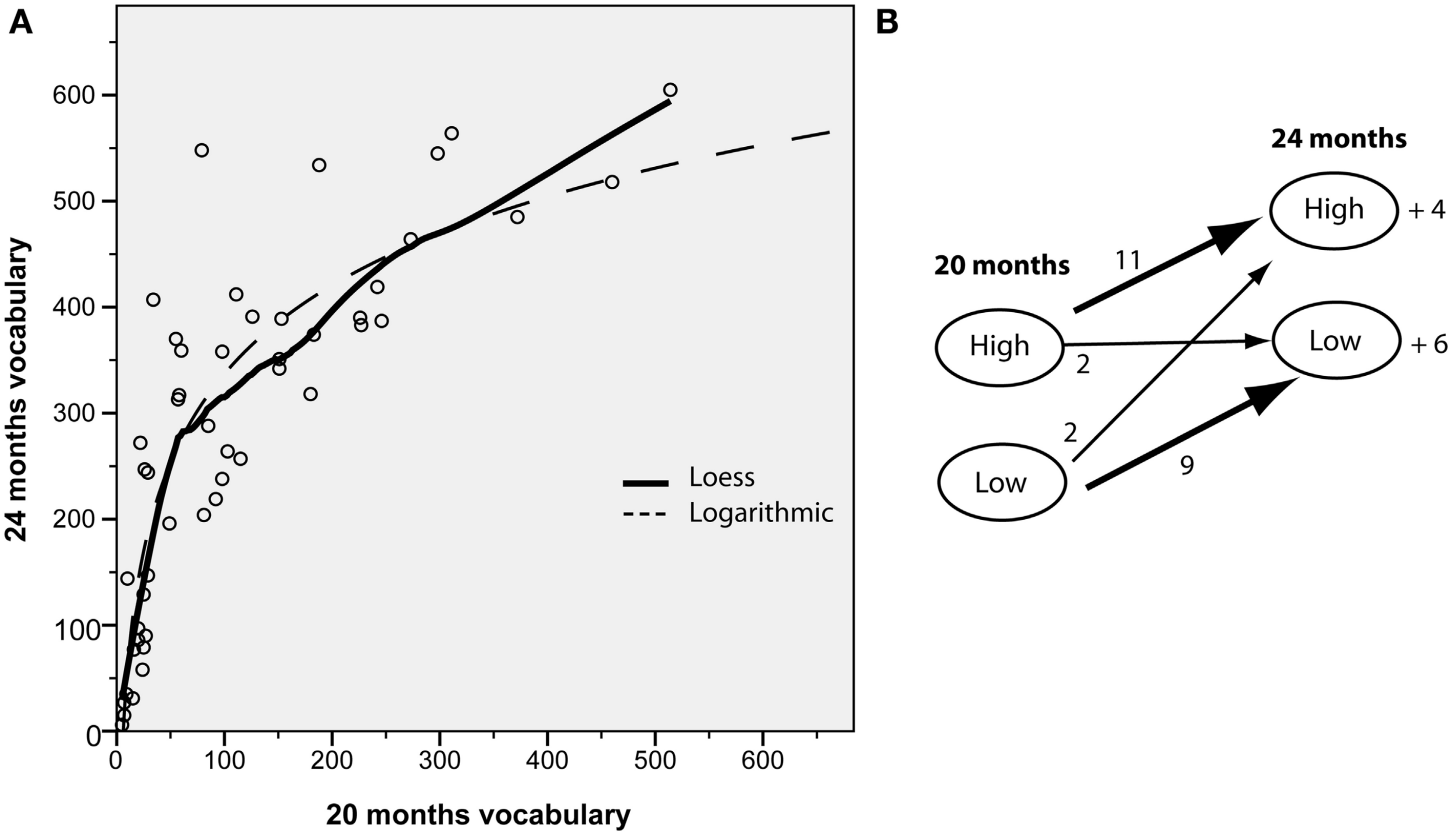
**(A)** Relation between productive vocabularies measured by the SECDI at 20 and 24 months. The dotted line represents a logarithmic fitted line, and the connected line represents a loess fit. **(B)** Illustration of participants from the ERP samples included in the high vs. low vocabulary groups at the two time points. The number by the arrow represents the number of participants from the 20 month groups that were included in each group at 24 months. Note that 24 participants from the 20 month sample provided ERP data at 24 months, and 10 “novel” participants entered the 24 month groups.

We explored the relation between vocabulary size at 20 and 24 months in the overall sample (*n* = 49). In a simple linear regression, there was a strong positive correlation (β = 0.784, *p* < 0.001). It was clear from a scatter-plot of the two variables that vocabularies increased in a non-linear manner. A regression with a logarithmic estimation provided a better fit for the data (β = 0.877, *p* < 0.001), and this was confirmed by a loess fitted line (data-driven) that very closely followed the logarithmic fitted line (see Figure 7A). Small vocabularies at 20 months showed a greater increase by 24 months than larger vocabularies. Figure 7B shows the movement between vocabulary groups, used in the ERP analysis, at the two time points.

The longitudinal design permitted us to see if the ERP incongruity responses and behavioral performance at 20 months predicted later vocabulary at 24 months. The ERP measures used as predictors were the parietal difference in amplitude between 500 and 900 ms as an effect of incongruity in each picture condition. For the silhouette condition we also tested the central effect that was found to interact with 20 month productive vocabulary. These variables were entered into a step-wise multiple regression model, and the only variable to be included was the central silhouette effect (β = −0.357, *p* = 0.045). The other measures were all completely uncorrelated with 24 month vocabulary (all *r* < 0.100, *p* > 0.300). A similar regression was performed including the two behavioral performance measures at 20 months, and none was found to be a significant predictor (detail condition: *r* = 0.145, *p* = 0.451; silhouette condition: *r* = 0.263, *p* = 0.169).

## Discussion

An attenuation of N400 amplitude in response to a word following a congruous picture compared to an incongruous picture indicates that children were able to recognize the object and that this visual information activated semantic information and the associated lexical item. Different strengths of the N400 effect then suggest different levels of efficiency in the connection between the visual stimulus and associated word.

A critical control in this experiment was the semantic priming effect from regular whole pictures of common objects. Both 20-month-olds and 24-month-olds responded with an N400 incongruity effect that did not interact with vocabulary size. This replicates several previous studies demonstrating the presence of an N400 incongruity effect in this age group and younger children, in similar picture-word mapping paradigms (Friedrich and Friederici, 2004, 2005a, 2006, 2008, 2010; Torkildsen et al., 2006, 2008, 2009). The longitudinal comparison also showed that the N400 effect grew stronger with age.

### Word mapping based on shape information

The main hypothesis in this study concerned children's use of object shape information in word learning, and the role of shape recognition in vocabulary development. We expected the semantic priming effect by silhouette presentations to correlate with vocabulary size. In line with our hypothesis, the ERP results showed that children displayed different patterns of the incongruity effect in silhouette contexts depending on vocabulary size. Children with large vocabularies had a centro-parietal distribution of the N400, while those with small vocabularies only displayed a parietal effect. At 20 months, this pattern was directly correlated with individual vocabulary scores, where a larger vocabulary was associated with a larger incongruity effect over central electrode sites. This suggests that, although children at both levels of development were semantically primed by object silhouettes and responded to the incongruity, they processed the information in different ways, which was not the case in the regular picture condition. The results at 24 months can be seen as a replication of the 20 month results at the group level. Even with an addition of novel participants and some movement between vocabulary groups, the group differences remained. However, the lack of a correlation between the continuous vocabulary measure and ERP amplitude suggests a less direct relation.

A similar pattern of a more frontal distribution of the N400 in children with larger vocabularies was found in Torkildsen et al. (2008), although in that experiment only regular pictures of objects were used as stimuli. It is possible that the more central distribution in children with large vocabularies indicates involvement of additional processes related to enhanced attention and stimulus saliency, such as seen in the Nc component. This ERP component, observed only in infants and children (Csibra et al., 2008), is understood to reflect aspects of attention related to the perception of salient and interesting events (Courchesne, 1978). In the present experimental context, the large central negativity to words following incongruous silhouettes compared to congruous ones might indicate that the children with large vocabularies perceived this as a highly salient event and recruited more attentional resources, perhaps in an attempt to integrate the word in the incongruous context. This interpretation supports the idea that attention to shape properties of objects increases as early vocabularies reach a certain size (Pereira and Smith, 2009; Smith, 2009, 2013; Yee et al., 2012). At 20 months, when this correlation was significant, half of the sample had vocabularies under 80 words, which is around the common threshold of the vocabulary spurt (50–75 words), while at 24 months, when most of the children had vocabularies of at least a 100 words, there was no longer a significant relation between the incongruity effect and productive vocabulary. This indicates that modulations of the incongruity effect were influenced by whether children had particularly small vocabularies, and not just any differences in vocabulary size. Regardless of whether the acceleration in vocabulary growth is viewed as continuous or a “spurt,” the non-linear relation between 20 and 24 month vocabulary, which showed a steeper increase in vocabularies that were relatively small at 20 months while the growth rate leveled off for larger vocabularies, illustrates that the current sample underwent a growth pattern consistent with the onset of a vocabulary spurt before 100 words.

In contrast to previous behavioral studies on shape caricature recognition, which have used 3-dimensional object stimuli, the present study used 2-dimensional cartoon images of objects. This enabled a computerized stimulus presentation, but also an extension of the current knowledge about which type of shape cues that have a special role in word learning. Our shape condition used outline shape in the form of a silhouette, rather than an abstract 3-dimensional shape caricature. It has been demonstrated that adults easily recognize objects from outline images, and that outline shapes function as primes for object labels (Rosch et al., 1976; Hayward, 1998). Hayward (1998) theorized that “outline shape might be particularly crucial for achieving object constancy or recognition across a change in observed viewpoint,” which suggests that it may be an important feature in generalization in a way similar to how abstract shape caricatures are thought to promote generalization (e.g., Smith, 2009). Moreover, research has shown that toddlers and adults prefer an upright planar view-point when freely handling and viewing three-dimensional objects (Pereira et al., 2010), which suggests that using two-dimensional planar views of objects is appropriate when studying children's object recognition.

Our behavioral experiment used the same type of stimuli as the ERP experiment, but the task required explicit identification of objects corresponding to the word used. Children performed this task relatively well at both ages, and interestingly, the ability to identify silhouette images correlated with the parietal ERP incongruity effect at 20 months, but not at 24 months. Thus, 20-month-olds who were better at identifying word referents from silhouettes had a stronger parietal N400 incongruity effect. This correlation between our two experimental tasks supports our interpretation of the ERP incongruity effects as reflecting referential lexical processing. The fact that there was no such correlation at 24 months suggests that at that age most children's N400 responses had matured to the point where the efficient mechanism was in place and this electrophysiological response no longer accounted for the differences in behavioral performance. The differences in behavioral performance at 20 months, however, seem to be in part explained by weaker associations between words and silhouette versions of their referents, as indicated by the weaker N400 incongruity effect.

The correlation between performance in the behavioral experiment and vocabulary at 24 months but not at 20 months differs from earlier research which has found that shape caricature recognition correlates with vocabulary between 18 and 24 months (Pereira and Smith, 2009; Yee et al., 2012). Silhouette recognition specifically, however, was associated with other more general aspects of communicative development at 20 months, as measured by the ASQ Communication Scale which includes items about comprehension, the ability to point to objects when a parent asks for them, as well as word production. The difference in results regarding vocabulary compared to previous studies may be due to differences in stimuli, where our silhouette stimuli displayed a different kind of shape information than the three-dimensional abstract shape caricatures used previously. In relation to these earlier studies, outline shape seemed to be easier for children to recognize, judging by the ceiling effect seen in our data. This may be because outlines reveal more local information, and information about object parts, despite the lack of any surface details. By using a different type of stimulus material we can extend previous knowledge by showing that differential sensitivity to shape in naming contexts by children at different levels of vocabulary acquisition is not restricted to abstract shape caricatures, but also regards outline shape in two-dimensional images. For this type of stimuli, however, electrophysiological measures were able to capture this differential processing at an earlier age than behavioral measures.

In the present study, behavioral identification of both silhouette and detail pictures correlated with vocabulary at 24 months, but the correlation was stronger for the silhouette condition. This reveals an interesting pattern. Although the electrophysiological data suggest that children with larger vocabularies at 20 months attended more to words in silhouette contexts than children with smaller vocabularies, other factors than their language competence seemed to significantly affect their behavioral performance at this early age. Such factors could be related to social skills and mastering the general task of pointing to pictures. A difference between the ERP task and the behavioral task is that the ERP task did not require any active participation from the child, or interaction with an experimenter. In contrast, the behavioral task required interaction, and the ability to follow instructions, and perhaps individual differences in such abilities or motivations to participate contributed more to the differences in behavioral performance. The ERP measures on the other hand could more directly pick up differences in cognitive processes related to language skills. It is also possible that the children who were able to perform the behavioral task, after a demanding ERP experiment, as a group had higher general cognitive skills (language and/or attention), and thus the variability was decreased in this group. This interpretation is supported by the significantly larger vocabulary size among the 20-month-olds who successfully performed the behavioral task. Four months later, when vocabularies had grown above a certain point, there was no systematic relation between individual vocabularies and ERP responses (although there were still group differences). But at this point, vocabulary was a more reliable predictor for behavioral performance. The attrition in the behavioral experiment was also much lower than at 20 months, and language skills in the behavioral sample more closely matched those of the entire sample.

Among all our experimental measures at 20 months, only the silhouette ERP incongruity effect predicted vocabulary size at 24 months. This supports the theory that sensitivity to shape information in relation to word labeling has particular relevance for vocabulary development. Since the N400 effect to regular pictures was independent of vocabulary size, we know that the effect of vocabulary size is not due to differences in the N400 mechanism. Rather, children with large vocabularies were more strongly semantically primed by object shape specifically. However, we do not know whether this stronger effect was due to a richer semantic understanding of the specific words used, or whether it was driven by a generally larger vocabulary size. The extensive familiarization phase with five congruous pairings was incorporated in order to minimize differences in specific stimulus familiarity, and this has been shown to make a critical difference. When using a familiarization phase even 9-month-old infants can produce N400 effects to words (Junge et al., 2012), while only 12-month-olds with large vocabularies showed the effect when a familiarization phase was not present (Friedrich and Friederici, 2011). One study that attempted to test the hypothesis that the N400 effect is a reliable measure of specific vocabulary knowledge, found no correlation between the N400 effect and behavioral measures of vocabulary knowledge for the same items in 8- to 9-year-old children. Instead, the N400 correlated with general listening comprehension (Henderson et al., 2011). It is still possible that a behavioral measure of vocabulary knowledge does not pick up subtle differences in semantic knowledge of specific items, or simply the efficiency with which words are retrieved from long-term memory upon encounter with different semantic cues. Such differences in efficiency may be due to both experience with specific words and general experience with linking words and their referents which comes with a larger vocabulary. It is likely that these two factors are both involved in the differences in N400 effects seen in children with different vocabulary sizes.

### Word mapping to object parts

Another of our main hypotheses was that children progress from object recognition based on fragments to that based on shape, which has been suggested by some previous experiments and theories (Pereira and Smith, 2009; Smith, 2009, 2013). We aimed to test this more directly than previous studies, by using stimuli displaying detached object parts rather than parts connected with an incorrect configuration. Our results did not provide any evidence for this hypothesis. First, behavioral performance in the detail condition was worse than the silhouette condition for all children at both 20 and 24 months, and correlated positively with vocabulary at 24 months. In contrast, Pereira and Smith (2009) found that children with the smallest vocabularies were better at recognizing objects from only parts information. Also, we found no ERP evidence of semantic priming from the detail pictures at either age, which would be expected if children recognized the objects from the pictures and mapped them to known word representations. Visual inspection of the ERP waveforms at 24 months, and the lack of a significant interaction between congruity and picture condition at this age, suggests that the processing of the part-object stimuli was becoming more similar to the processing seen in the other conditions, which could mean that the children may be semantically primed by object fragments when they grow older. However, our results suggest that children do not recognize objects based on isolated fragments early in development. It has been reliably demonstrated that children have a whole-object bias when mapping words to objects, which means that they assume that words refer to whole objects and not parts of objects (e.g., Markman, 1990, 1992). Therefore, it is possible that children did not perceive the detail pictures in the present study as whole objects, and thus the whole object word was not primed by the picture. Rather, perhaps words referring to the object parts such as “eye,” “door,” “leg” etc. were activated by the picture presentation. While there was a lack of priming by detail pictures in the ERP task, the behavioral task showed that the children were able to recognize the objects in the detail pictures, although performance was clearly lower than in the silhouette task, indicating that many had difficulty identifying these stimuli. This discrepancy may be due to the relatively fast presentation rate in the ERP task compared to the behavioral task where there was no time limit. Moreover, the behavioral task was a forced choice between two items and could be solved by simply choosing the picture that was the best match for the word. The lack of ERP effects in the detail condition and the relatively poor performance in identifying the part-objects behaviorally suggest that the stimuli used in the present study engage other processes of object recognition than the part-object stimuli used in Pereira and Smith (2009). Consequently, it would be interesting to see future studies using two-dimensional object drawings that lack shape information but still keep object parts unified. Such an approach would require that one determines which features that constitute a part, and then move these around in different constellations. It is possible that such stimuli would function better as word primes, and if so they should also be easier to identify behaviorally. In preparing the present study, such stimuli were considered, but proved difficult to create while still maintaining a reasonable level of recognizability.

Given the uniqueness of the stimuli used in this experiment, our results do not allow any conclusions about the accuracy of the theory about object recognition progressing from non-configural to shape-based. However, the term “fragment”-based has been used to describe the early form of object recognition (Smith, 2009). Our results speak against this interpretation, because the object fragments used in the present study were found to be more difficult to recognize than object silhouettes. A possible argument against this conclusion is that children may need *all* object fragments to be present, and our results would be due to the fact that only a few salient parts were displayed. In that case, it would mean that children take into account the combination of parts, rather than individual fragments. We can, however, conclude that children found it more difficult to identify objects from pictures of fragments than from silhouettes, regardless of their stage of vocabulary development.

## Conclusions

We conducted a longitudinal study to investigate the link between toddlers' sensitivity to object shape vs. object parts in labeling contexts, for the first time linking behavioral and electrophysiological measures of word-object mapping in this research area. We found that, in 20-month-olds, the N400 incongruity effect to words primed by silhouette pictures varied in topography and amplitude depending on vocabulary size, and these differences predicted the children's productive vocabulary size 4 months later. ERP responses elicited in regular picture contexts and detail picture contexts were not related to vocabulary size. At 24 months, electrophysiological responses to shape did not correlate with vocabulary size. By comparing ERP responses with a behavioral test of object recognition, we could see that our ERP measure of word-shape mapping correlated with vocabulary size already at 20 months, while behavioral performance did not correlate with vocabulary until 24 months. This highlights the methodological strength of combining different measures, and the added sensitivity provided by electrophysiological measures in picking up individual differences. In sum, we have demonstrated that productive vocabulary size is related to differences in electrophysiological priming of words by object shape, around the developmental time point of the vocabulary spurt.

### Conflict of interest statement

The authors declare that the research was conducted in the absence of any commercial or financial relationships that could be construed as a potential conflict of interest.

## Footnotes

^1^*Clipart*. Vital Imagery Ltd. Clipart Available online at: http://www.clipart.com [Accessed 2009].

## References

[B1] American Heritage Dictionary. (2004). Fragment, in The American Heritage Dictionary of the English Language. 5th Edn Available online at: http://www.ahdictionary.com/word/search.html?q=fragment

[B2] Atchley R. A. Rice M. L. Betz S. K. Kwasny K. M. Sereno J. A. Jongman A. (2006). A comparison of semantic and syntactic event related potentials generated by children and adults. Brain Lang. 99, 236–246. 10.1016/j.bandl.2004.04.003 16226804

[B3] Augustine E. Smith L. B. Jones S. S. (2011). Parts and relations in young children's shape-based object recognition. J. Cogn. Dev. 12, 556–572. 10.1080/15248372.2011.560586 24285930 PMC3840158

[B4] Bion R. A. H. Borovsky A. Fernald A. (2013). Fast mapping, slow learning: disambiguation of novel word–object mappings in relation to vocabulary learning at 18, 24, and 30 months. Cognition 126, 39–53. 10.1016/j.cognition.2012.08.008 23063233 PMC6590692

[B5] Colunga E. Smith L. B. (2008). Knowledge embedded in process: the self-organization of skilled noun learning. Dev. Sci. 11, 195–203. 10.1111/j.1467-7687.2007.00665.x 18333974

[B6] Courchesne E. (1978). Neurophysiological correlates of cognitive development: changes in long-latency event-related potentials from childhood to adulthood. Electroencephalogr. Clin. Neurophysiol. 45, 468–482. 10.1016/0013-4694(78)90291-2 81749

[B6a] Csibra G. Kushnerenko E. Grossmann T. (2008). Electrophysiological methods in studying infant cognitive development, in Handbook of Developmental Cognitive Neuroscience, eds Nelson C. Luciana M. (Cambridge: MIT Press), 247–262.

[B7] Delorme A. Makeig S. (2004). EEGLAB: an open source toolbox for analysis of single-trial EEG dynamics including independent component analysis. J. Neurosci. Methods 134, 9–21. 10.1016/j.jneumeth.2003.10.009 15102499

[B8] Dick F. Leech R. Richardson F. (2008). The neuropsychology of language development, in Child Neuropsychology: Concepts, Theory and Practice, eds Reed J. Warner-Rogers J. (West Sussex: Wiley-Blackwell), 139–182.

[B9] Eriksson M. Berglund E. (2002). Instruments, Scoring Manual and Percentile Levels of the Swedish Early Communicative Development Inventory, SECDI (FoU-rapport 17th Edn). Gävle: Institutionen för pedagogik, didaktik och psykologi.

[B10] Fenson L. Dale P. S. Reznick J. S. Bates E. Thal D. J. Pethick S. J. (1994). Variability in early communicative development. Monogr. Soc. Res. Child Dev. 59, 1–173. discussion: 174–185. 7845413

[B9a] Fenson L. Dale P. S. Reznick J. S. Thal D. Bates E. Hartung J. (1993). The MacArthur Communicative Development Inventories: User's Guide and Technical Manual, San Diego, CA: Singular Publisher.

[B10a] Friedrich M. (2011). Early word learning: reflections on behavior, connectionist models, and brain mechanisms indexed by ERP components, in The Handbook of Psycholinguistic and Cognitive Processes: Perspectives in Communication Disorders, eds Guendouzi J. Loncke F. Williams M. J. (New York, NY: Psychology Press), 145–188.

[B11] Friedrich M. Friederici A. (2004). N400-like semantic incongruity effect in 19-month- olds: processing known words in picture contexts. J. Cogn. Neurosci. 16, 1465–1477. 10.1162/0898929042304705 15509391

[B12] Friedrich M. Friederici A. (2005a). Lexical priming and semantic integration reflected in the event-related potential of 14-month-olds. Neuroreport 16, 653–656. 10.1097/00001756-200504250-00028 15812327

[B13] Friedrich M. Friederici A. (2005b). Phonotactic knowledge and lexical-semantic processing in one-year-olds: brain responses to words and nonsense words in picture contexts. J. Cogn. Neurosci. 17, 1785–1802. 10.1162/089892905774589172 16269114

[B14] Friedrich M. Friederici A. (2006). Early N400 development and later language acquisition. Psychophysiology 43, 1–12. 10.1111/j.1469-8986.2006.00381.x 16629680

[B15] Friedrich M. Friederici A. (2008). Neurophysiological correlates of online word learning in 14-month-old infants. Neuroreport 19, 1757–1761. 10.1097/WNR.0b013e328318f014 18955904

[B16] Friedrich M. Friederici A. (2010). Maturing brain mechanisms and developing behavioral language skills. Brain Lang. 114, 66–71. 10.1016/j.bandl.2009.07.004 19665783

[B17] Friedrich M. Friederici A. (2011). Word learning in 6-month-olds: fast encoding-weak retention. J. Cogn. Neurosci. 23, 3228–3240. 10.1162/jocn_a_00002 21391764

[B18] Ganger J. Brent M. R. (2004). Reexamining the vocabulary spurt. Dev. Psychol. 40, 621–632. 10.1037/0012-1649.40.4.621 15238048

[B19] Gershkoff-Stowe L. Smith L. B. (2004). Shape and the first hundred nouns. Child Dev. 75, 1098–1114. 10.1111/j.1467-8624.2004.00728.x 15260867

[B20] Hayward W. G. (1998). Effects of outline shape in object recognition. J. Exp. Psychol. Hum. Percept. Perform. 24, 427–440 10.1037//0096-1523.24.2.427

[B21] Henderson L. M. Baseler H. A. Clarke P. J. Watson S. Snowling M. J. (2011). The N400 effect in children: relationships with comprehension, vocabulary and decoding. Brain Lang. 117, 88–99. 10.1016/j.bandl.2010.12.003 21272930

[B22] Holcomb P. J. Coffey S. A. Neville H. J. (1992). Visual and auditory sentence processing: a developmental analysis using event-related brain potentials. Dev. Neuropsychol. 8, 203–241 10.1080/87565649209540525

[B23] Horst J. S. Samuelson L. K. (2008). Fast mapping but poor retention by 24-month-old infants. Infancy 13, 128–157 10.1080/15250000701795598 33412722

[B24] Horst J. S. Scott E. J. Pollard J. A. (2010). The role of competition in word learning via referent selection. Dev. Sci. 13, 706–713. 10.1111/j.1467-7687.2009.00926.x 20712736

[B25] Janson H. Smith L. (2003). Norsk Manualsupplement til Ages and Stages Questionnaires. Oslo: Regionsenter for barn og unges psykiske helse, Helseregion Øst/Sør.

[B26] Jones S. S. Smith L. B. (2005). Object name learning and object perception: a deficit in late talkers. J. Child Lang. 32, 223–240. 10.1017/s0305000904006646 15779885

[B27] Junge C. Cutler A. Hagoort P. (2012). Electrophysiological evidence of early word learning. Neuropsychologia 50, 3702–3712. 10.1016/j.neuropsychologia.2012.10.012 23108241

[B28] Juottonen K. Revonsuo A. Lang H. (1996). Dissimilar age influences on two ERP waveforms (LPC and N400) reflecting semantic context effect. Cogn. Brain Res. 4, 99–107. 10.1016/0926-6410(96)00022-5 8883923

[B29] Kutas M. Federmeier K. D. (2011). Thirty years and counting: finding meaning in the N400 component of the event-related brain potential (ERP). Annu. Rev. Psychol. 62, 621–647. 10.1146/annurev.psych.093008.131123 20809790 PMC4052444

[B28a] Kutas M. Hillyard S. A. (1980). Reading senseless sentences: brain potentials reflect semantic incongruity. Science 207, 203–205. 7350657 10.1126/science.7350657

[B30] Landau B. Smith L. B. Jones S. S. (1988). The importance of shape in early lexical learning. Cogn. Dev. 3, 299–321 10.1016/0885-2014(88)90014-7

[B30a] Lau E. F. Phillips C. Poeppel D. (2008). A cortical network for semantics: (De)constructing the N400. Nat. Rev. Neurosci. 9, 920–933. 10.1038/nrn2532 19020511

[B31] Lopez-Calderon J. Luck S. J. (2014). ERPLAB: an open-source toolbox for the analysis of event-related potentials. Front. Hum. Neurosci. 8:213. 10.3389/fnhum.2014.00213 24782741 PMC3995046

[B32] Markman E. M. (1990). Constraints children place on word meanings. Cogn. Sci. 14, 57–77 10.1207/s15516709cog1401_4

[B33] Markman E. M. (1992). Constraints on word learning: speculations about their nature, origins and domain specificity, in Modularity and Constraints in Language and Cognition: The Minnesota Symposium on Child Psychology, Vol. 25, eds Gunnar M. R. Maratsos M. P. (Hillsdale, NJ: Erlbaum), 59–101.

[B34] Mayor J. Plunkett K. (2010). A neurocomputational account of taxonomic responding and fast mapping in early word learning. Psychol. Rev. 117, 1–31. 10.1037/a0018130 20063962

[B35] McMurray B. (2007). Defusing the childhood vocabulary explosion. Science 317, 1503–1523. 10.1126/science.1144073 17673655

[B36] Mills D. L. Plunkett K. Prat C. Schafer G. (2005). Watching the infant brain learn words: effects of vocabulary size and experience. Cogn. Dev. 20, 19–31 10.1016/j.cogdev.2004.07.001

[B37] Nazzi T. Bertoncini J. (2003). Before and after the vocabulary spurt: two modes of word acquisition? Dev. Sci. 6, 136–142 10.1111/1467-7687.00263

[B38] Pereira A. F. James K. H. Jones S. S. Smith L. B. (2010). Early biases and developmental changes in self-generated object views. J. Vis. 10, 22–22. 10.1167/10.11.22 20884517 PMC3049954

[B39] Pereira A. F. Smith L. B. (2009). Developmental changes in visual object recognition between 18 and 24 months of age. Dev. Sci. 12, 67–83. 10.1111/j.1467-7687.2008.00747.x 19120414 PMC2888029

[B40] Perry L. K. Samuelson L. K. (2011). The shape of the vocabulary predicts the shape of the bias. Front. Psychol. 2:345. 10.3389/fpsyg.2011.00345 22125547 PMC3222225

[B41] Perry L. K. Samuelson L. K. Malloy L. M. Schiffer R. N. (2010). Learn locally, think globally: exemplar variability supports higher-order generalization and word learning. Psychol. Sci. 21, 1894–1902. 10.1177/0956797610389189 21106892 PMC3144952

[B42] Picton T. W. Bentin S. Berg P. Donchin E. Hillyard S. A. Johnson R. Jr.. (2000). Guidelines for using human event-related potentials to study cognition: recording standards and publication criteria. Psychophysiology 37, 127–152. 10.1017/s0048577200000305 10731765

[B43] Poulin-Dubois D. Frank I. Graham S. A. Elkin A. (1999). The role of shape similarity in toddlers' lexical extensions. Br. J. Dev. Psychol. 17, 21–36 10.1348/026151099165131

[B44] Rakison D. H. Butterworth G. E. (1998). Infants' use of object parts in early categorization. Dev. Psychol. 34, 49–62. 10.1037//0012-1649.34.1.49 9471004

[B45] Rämä P. Sirri L. Serres J. (2013). Development of lexical–semantic language system: N400 priming effect for spoken words in 18- and 24-month old children. Brain Lang. 125, 1–10. 10.1016/j.bandl.2013.01.009 23435193

[B46] Rosch E. Mervis C. B. Gray W. D. Johnson D. M. Boyes-Braem P. (1976). Basic objects in natural categories. Cogn. Psychol. 8, 382–439 10.1016/0010-0285(76)90013-x

[B47] Schafer G. Plunkett K. (1998). Rapid word learning by fifteen-month-olds under tightly controlled conditions. Child Dev. 69, 309–320. 10.2307/1132166 9586207

[B48] Smith L. B. (2003). Learning to recognize objects. Psychol. Sci. 14, 244–250 10.1111/1467-9280.03439 12741748

[B49] Smith L. B. (2009). From fragments to geometric shape: changes in visual object recognition between 18 and 24 months. Curr. Dir. Psychol. Sci. 18, 290–294. 10.1111/j.1467-8721.2009.01654.x 32489232 PMC7265591

[B50] Smith L. B. (2013). It's all connected: pathways in visual object recognition and early noun learning. Am. Psychol. 68, 618–629. 10.1037/a0034185 24320634 PMC3858855

[B51] Smith L. B. Colunga E. Yoshida H. (2010). Knowledge as process: contextually cued attention and early word learning. Cogn. Sci. 34, 1287–1314. 10.1111/j.1551-6709.2010.01130.x 21116438 PMC2992382

[B52] Smith L. B. Jones S. S. Landau B. Gershkoff-Stowe L. Samuelson L. (2002). Object name learning provides on-the-job training for attention. Psychol. Sci. 13, 13–19. 10.1111/1467-9280.00403 11892773

[B53] Son J. Y. Smith L. B. Goldstone R. L. (2008). Simplicity and generalization: short-cutting abstraction in children's object categorizations. Cognition 108, 626–638. 10.1016/j.cognition.2008.05.002 18565504 PMC2584368

[B54] Squires J. Potter L. Bricker D. (1999). The ASQ User's Guide for the Ages and Stages Questionnaires: A Parent-Completed, Child-Monitoring System, 2nd Edn Baltimore: Paul H. Brookes Publishing Co.

[B55] Torkildsen J. V. K. Friis Hansen H. Svangstu J. M. Smith L. Simonsen H. G. Moen I.. (2009). Brain dynamics of word familiarization in 20-month-olds: effects of productive vocabulary size. Brain Lang. 108, 73–88. 10.1016/j.bandl.2008.09.005 18950850

[B56] Torkildsen J. V. K. Sannerud T. Syversen G. Thormodsen R. Simonsen H. G. Moen I. (2006). Semantic organization of basic-level words in 20-month-olds: an ERP study. J. Neurolinguistics 19, 431–454 10.1016/j.jneuroling.2006.01.002

[B57] Torkildsen J. V. K. Svangstu J. M. Friis Hansen H. Smith L. Simonsen H. G. Moen I.. (2008). Productive vocabulary size predicts event-related potential correlates of fast mapping in 20-month-olds. J. Cogn. Neurosci. 20, 1266–1282. 10.1162/jocn.2008.20087 18284350

[B58] Torkildsen J., V. K. Syversen G. Simonsen H. G. Moen I. Lindgren M. (2007). Brain responses to lexical-semantic priming in children at-risk for dyslexia. Brain Lang. 102, 243–261. 10.1016/j.bandl.2006.11.010 17239944

[B59] Werker J. F. Cohen L. B. Lloyd V. L. Casasola M. Stager C. L. (1998). Acquisition of word-object associations by 14-month-old infants. Dev. Psychol. 34, 1289–1309. 10.1037/0012-1649.34.6.1289 9823513

[B60] Woodward A. L. Markman E. M. Fitzsimmons C. M. (1994). Rapid word learning in 13-month-olds and 18-month-olds. Dev. Psychol. 30, 553–566 10.1037//0012-1649.30.4.553

[B61] Yee M. Jones S. S. Smith L. B. (2012). Changes in visual object recognition precede the shape bias in early noun learning. Front. Psychol. 3:533. 10.3389/fpsyg.2012.00533 23227015 PMC3512352

[B62] Younger B. A. Cohen L. B. (1983). Infant perception of correlations among attributes. Child Dev. 54, 858–867. 10.2307/1129890 6617307

[B63] Younger B. A. Cohen L. B. (1986). Developmental change in infants' perception of correlations among attributes. Child Dev. 57, 803–815. 3720405

