# Supplementary material for: Event-related potentials during word mapping to object shape predict toddlers' vocabulary size
Source: Front Psychol. 2015 Feb 13;6:143. doi: 10.3389/fpsyg.2015.00143 (PMC4327527; doi:10.3389/fpsyg.2015.00143)
Supplement: Supplementary file 1 [file Image1.PDF]

Supplementary material 1

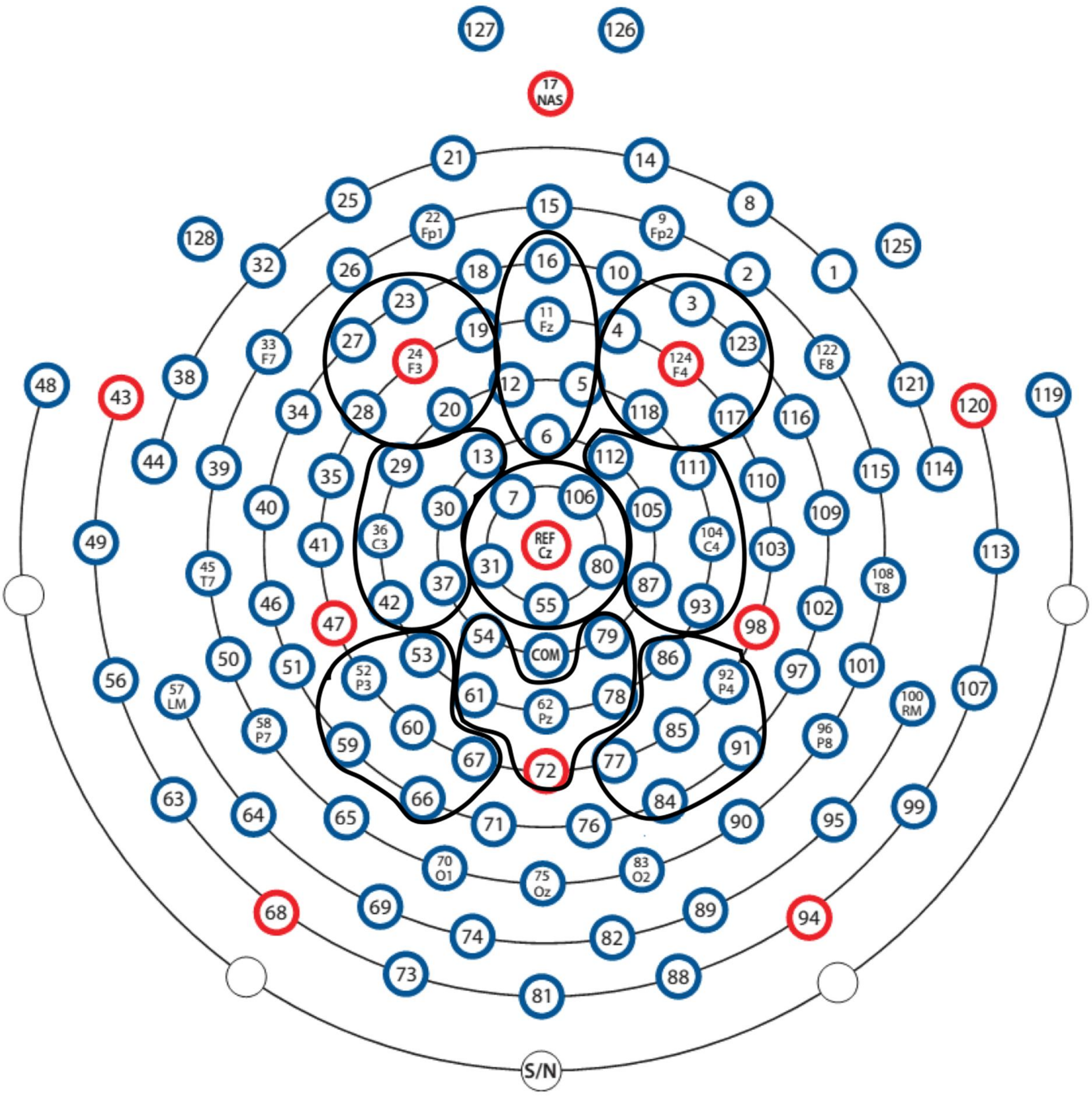

Channel layout of the Hydrocel Geodesic Sensor Net (128 channels). Channels selected for each of the 9 regions of interest have been circled.
