# Supplementary material for: Event-related potentials during word mapping to object shape predict toddlers' vocabulary size
Source: Front Psychol. 2015 Feb 13;6:143. doi: 10.3389/fpsyg.2015.00143 (PMC4327527; doi:10.3389/fpsyg.2015.00143)
Supplement: Supplementary file 2 [file Image2.PDF]

Supplementary material 2

Stimulus material in the ERP experiments

The ERP experiment at each time point (20 and 24 months) presented 30 common nouns (15 artifacts and 15 animals), and pictures of the corresponding referent. The words/pictures were selected with several factors in mind:

- that they were likely to be familiar to 20-24-month-olds
- that the object images had a clear outline shape
- that the object images had rich surface details that could be selected in the parts-condition

All pictures were different in the 20 and 24 months experiments, as well as most of the words. However, because of the challenge of selecting words and referents that fulfilled all the criteria above, 9 words were used at both 20 and 24 months. These are listed at the bottom of each of the stimulus tables, written in italics.

Stimulus material (words and pictures) used in the 20 month ERP experiment

**20 months**				
Word	***English translation***	***Regular***	***Silhouette***	***Detail***
bil	car	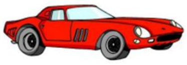	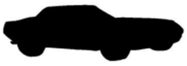	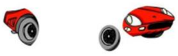
byxor	pants	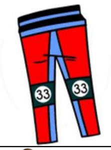	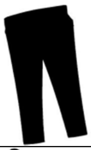	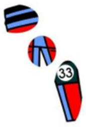
ekorre	squirrel	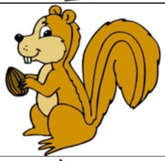	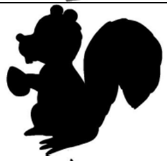	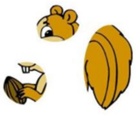
fisk	fish	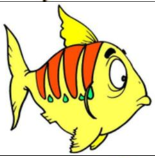	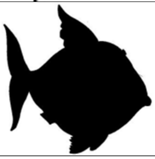	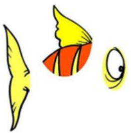
fjäril	butterfly	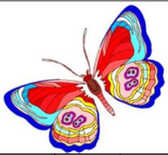	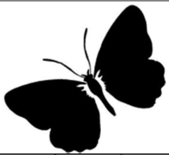	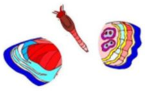
fluga	fly	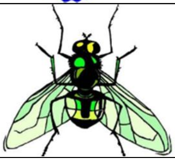	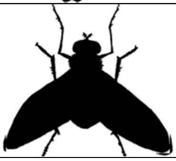	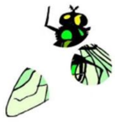

flygplan	airplane	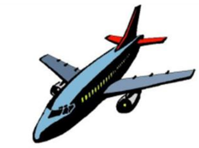	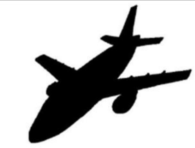	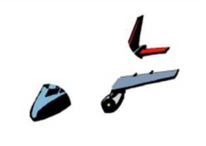
fågel	bird	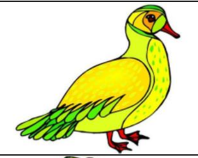	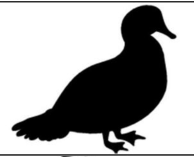	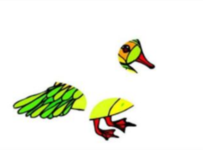
groda	frog	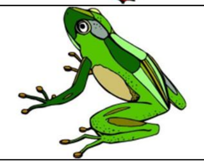	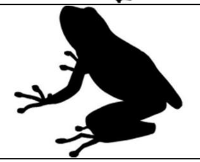	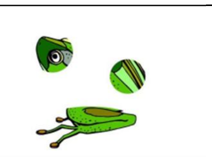
häst	horse	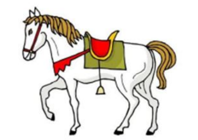	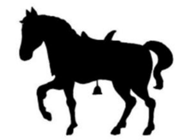	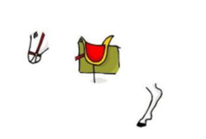
kossa	cow	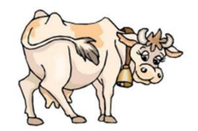	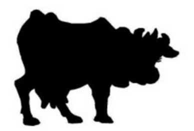	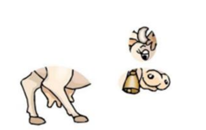
lejon	lion	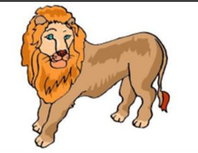	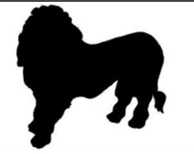	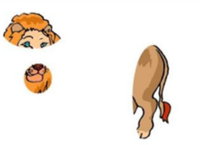
motorcykel	motorcycle	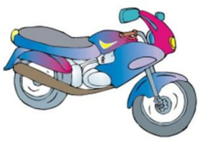	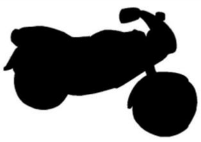	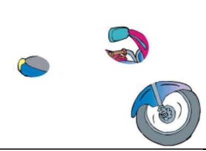
nalle	teddybear	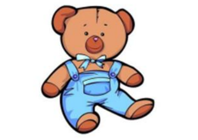	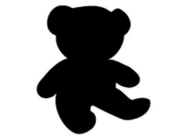	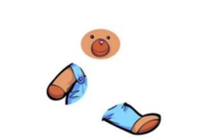
sko	shoe	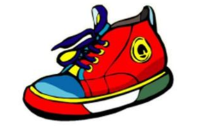	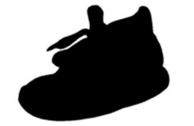	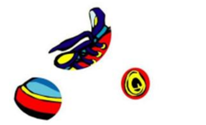
sköldpadda	turtle	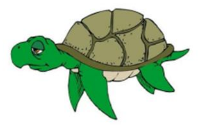	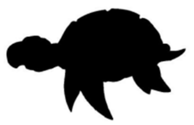	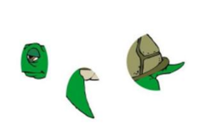
stol	chair	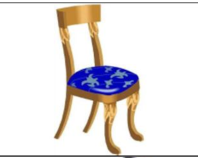	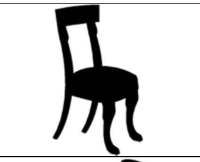	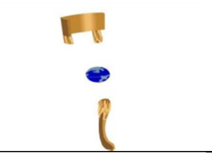
strumpa	sock	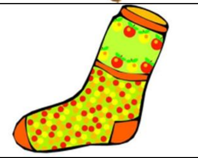	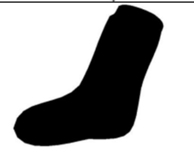	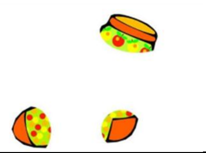

tandborste	toothbrush	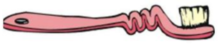	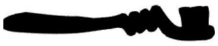	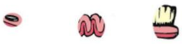
tupp	rooster	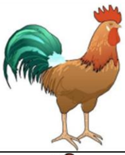	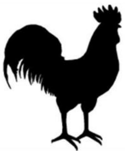	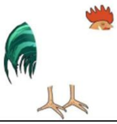
båt	*boat*	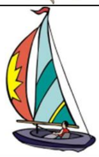	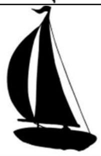	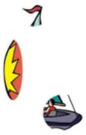
tåg	*train*	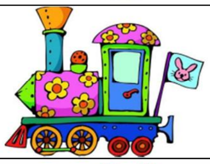	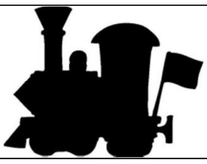	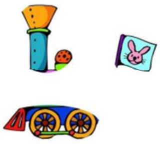
vagn	*stroller*	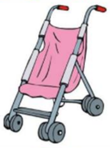	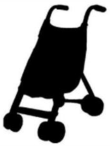	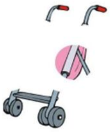
klänning	*dress*	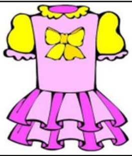	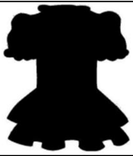	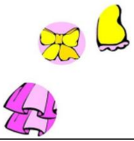
hund	*dog*	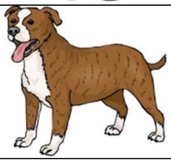	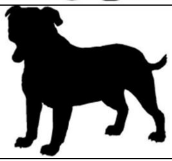	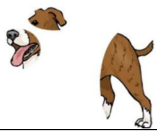
anka	*duck*	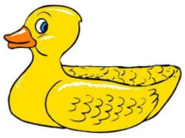	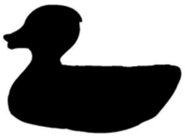	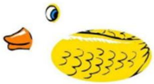
kanin	*rabbit*	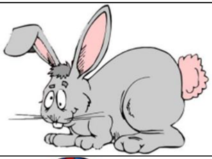	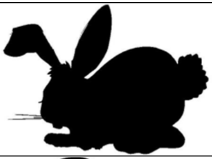	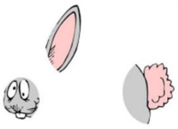
elefant	*elephant*	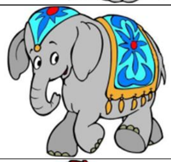	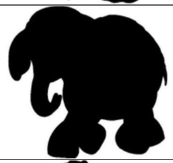	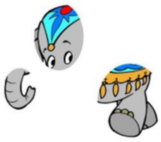
blomma	*flower*	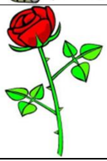	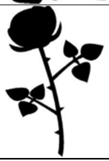	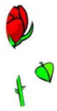

Stimulus material (words and pictures) used in the 24 months experiment

**24 months**				
Word	***English translation***	***Regular***	***Silhouette***	***Detail***
apa	monkey	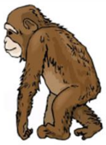	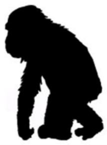	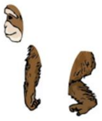
bi	bee	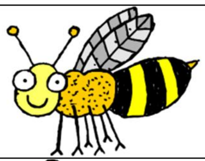	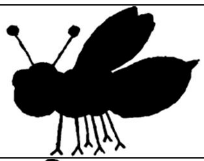	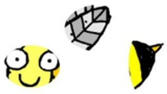
björn	bear	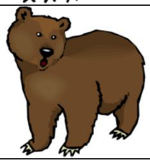	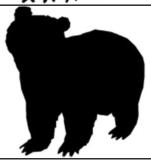	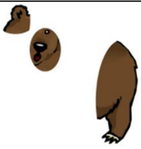
bord	table	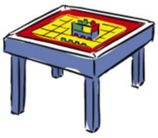	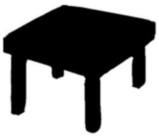	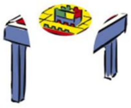
buss	bus	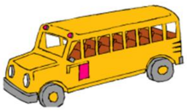	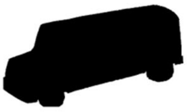	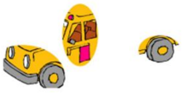
motorcykel	bicycle	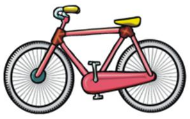	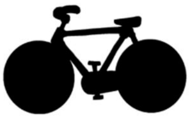	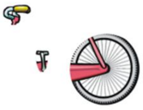
flaska	bottle	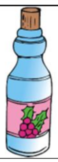	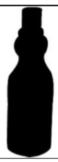	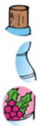
giraff	giraffe	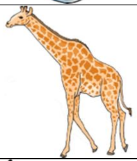	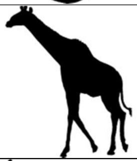	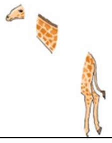
gris	pig	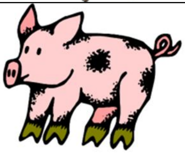	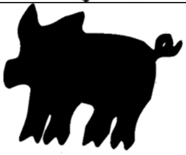	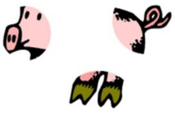
katt	cat	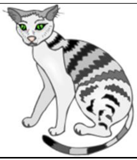	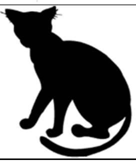	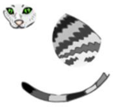

klocka	clock	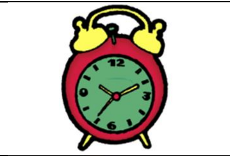	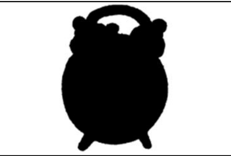	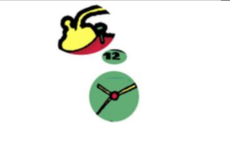
kyckling	chicken	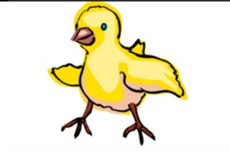	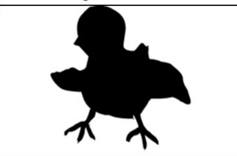	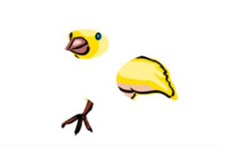
lamm	lamb	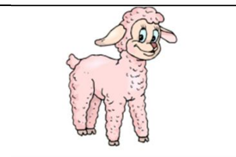	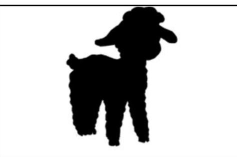	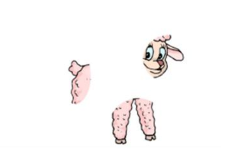
lampa	lamp	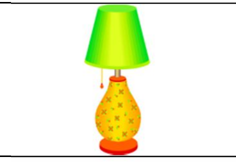	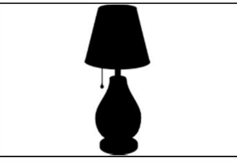	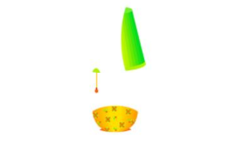
lastbil	truck	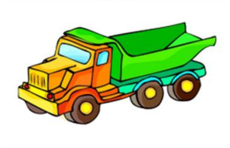	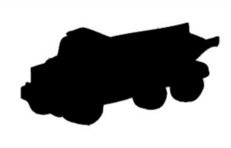	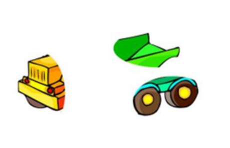
mus	mouse	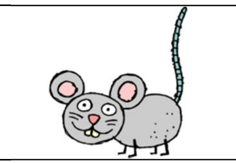	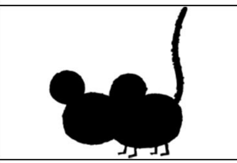	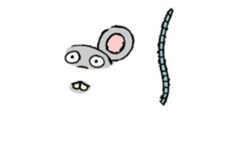
pingvin	penguin	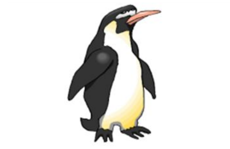	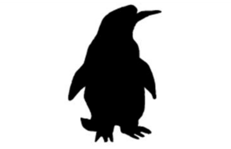	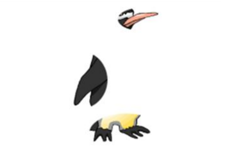
telefon	telephone	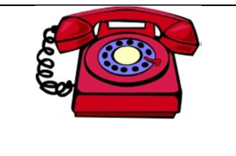	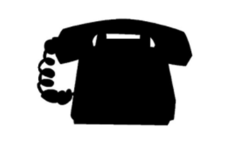	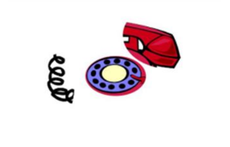
tiger	tiger	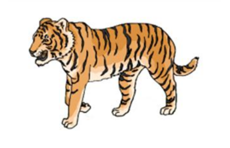	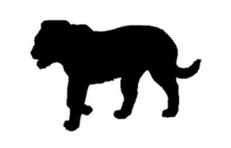	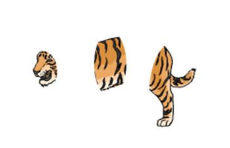
traktor	tractor	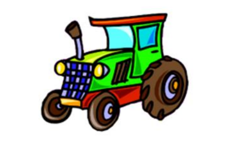	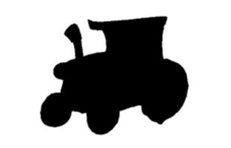	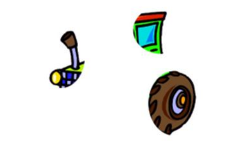
träd	tree	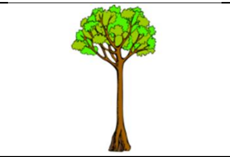	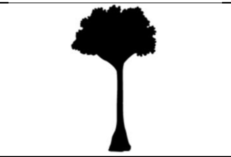	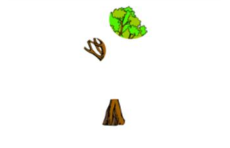
båt	*boat*	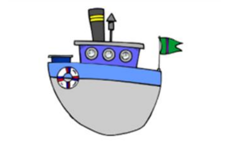	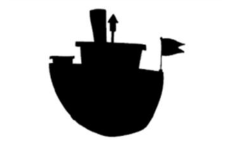	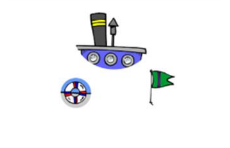

tåg	*train*	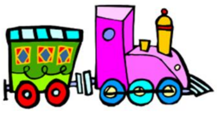	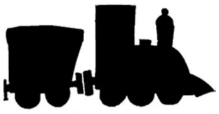	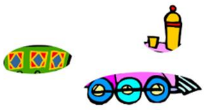
vagn	*stroller*	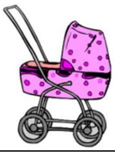	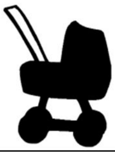	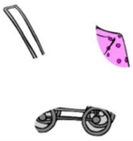
klänning	*dress*	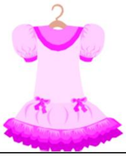	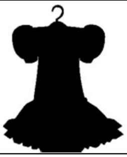	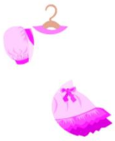
hund	*dog*	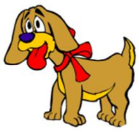	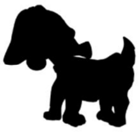	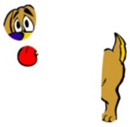
anka	*duck*	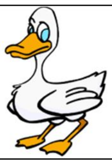	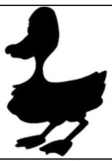	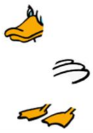
kanin	*rabbit*	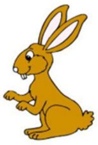	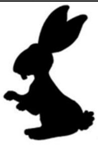	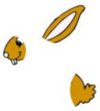
elefant	*elephant*	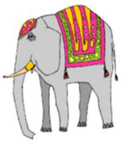	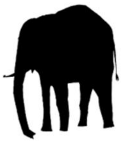	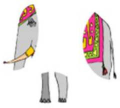
blomma	*flower*	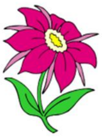	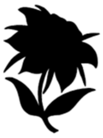	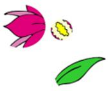
