# Supplementary material for: Event-related potentials during word mapping to object shape predict toddlers' vocabulary size
Source: Front Psychol. 2015 Feb 13;6:143. doi: 10.3389/fpsyg.2015.00143 (PMC4327527; doi:10.3389/fpsyg.2015.00143)
Supplement: Supplementary file 3 [file Image3.PDF]

Supplementary material 3

<u>Stimulus material in the behavioral experiments</u>

The stimulus nouns and their picture referents were selected according to the same criteria as in the ERP experiment.

At 20 months, the stimulus material for the behavioral experiment consisted of 18 nouns (9 artifacts and 9 animals) and corresponding pictures. At 24 months, the number of items was reduced to 14 (7 artifacts and 7 animals), in order to decrease attrition. All pictures differed at 20 and 24 months, and also differed from both ERP experiments. However, there was overlap in word items between the 20 and 24 months behavioral tests, and between the ERP and Behavioral experiments at each time point.

Stimulus material (words and pictures) used in the 20 months behavioral experiment

**20 months**				
Word	***English translation***	***Regular***	***Silhouette***	***Detail***
bil	Car	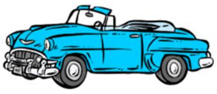	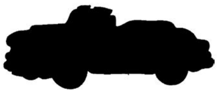	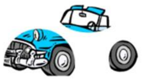
lampa	Lamp	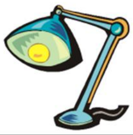	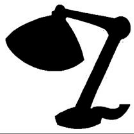	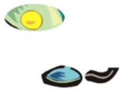
flygplan	Airplane	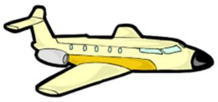	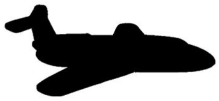	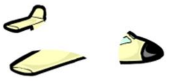
groda	Frog	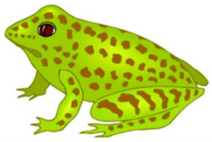	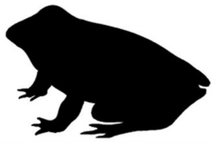	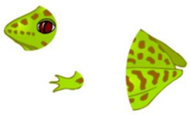
blomma	Flower	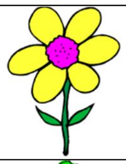	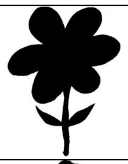	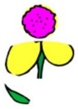
träd	Tree	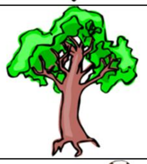	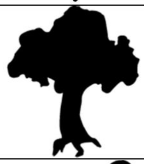	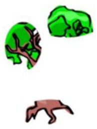
hund	Dog	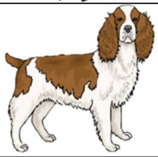	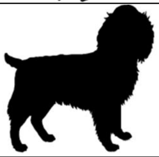	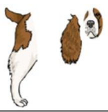

fisk	fish	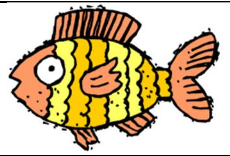	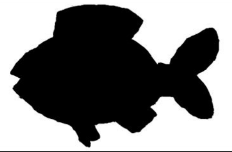	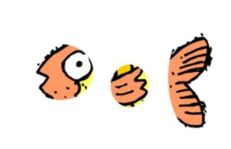
sköldpadda	Turtle	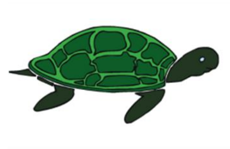	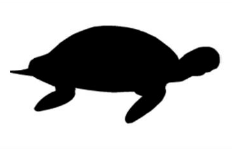	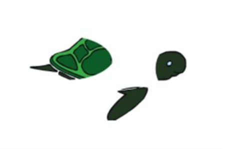
mus	Mouse	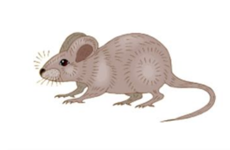	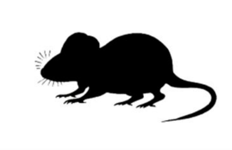	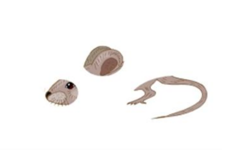
tåg	Train	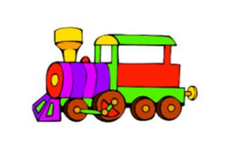	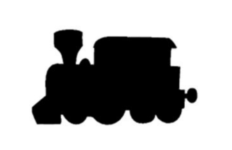	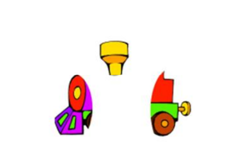
tröja	Shirt	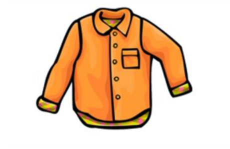	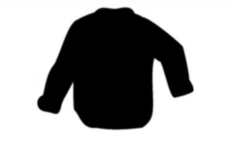	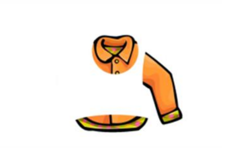
gris	Pig	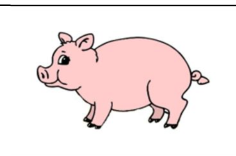	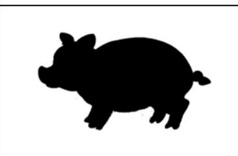	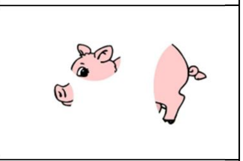
får	Sheep	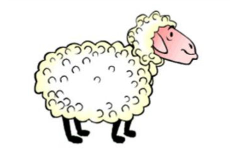	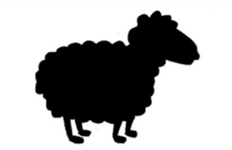	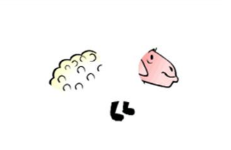
katt	Cat	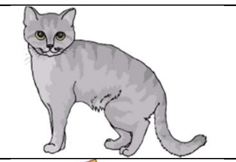	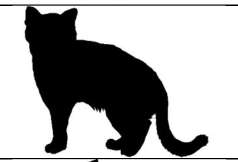	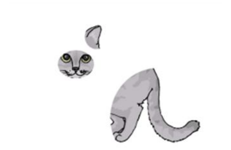
hus	House	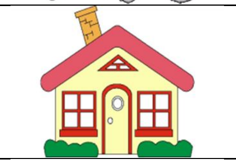	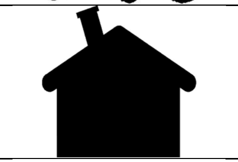	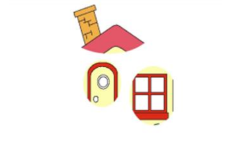
cykel	Bicycle	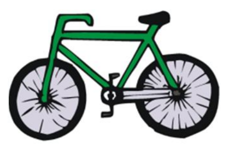	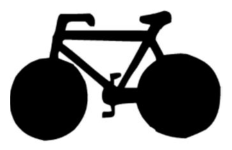	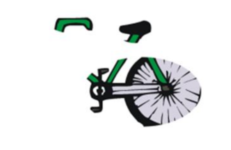
fjäril	butterfly	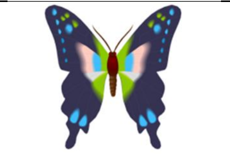	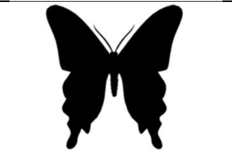	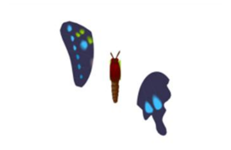

Stimulus material (words and pictures) used in the 24 months behavioral experiment

**24 months**				
Word	***English translation***	***Regular***	***Silhouette***	***Detail***
flaska	bottle	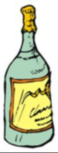	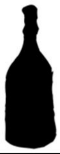	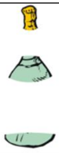
anka	duck	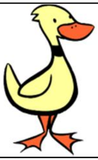	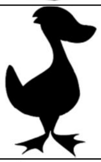	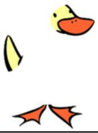
kanin	rabbit	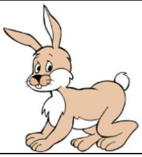	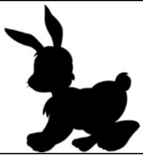	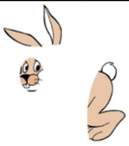
traktor	tractor	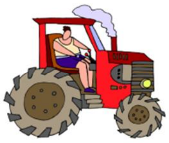	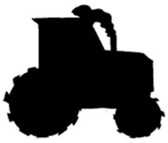	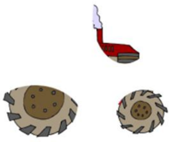
blomma	flower	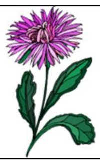	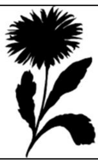	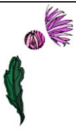
bi	bee	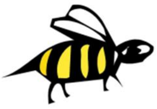	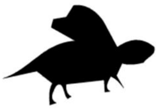	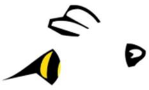
kyckling	chicken	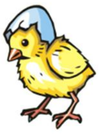	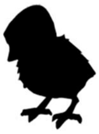	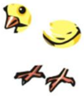
tiger	tiger	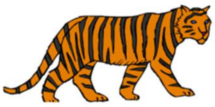	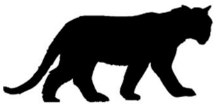	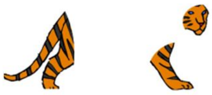
bord	table	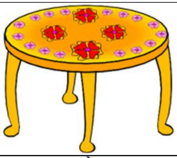	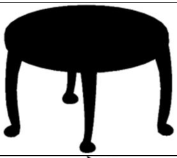	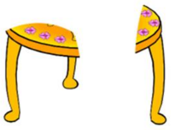
båt	boat	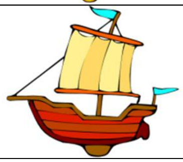	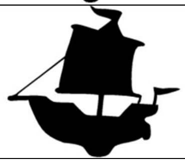	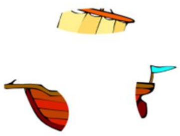

klänning	dress	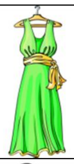	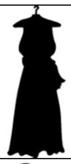	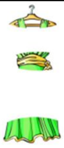
elefant	elephant	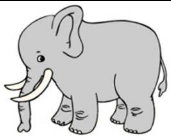	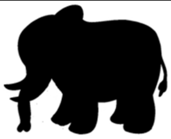	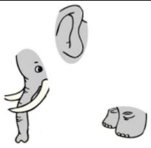
giraff	giraffe	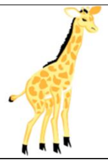	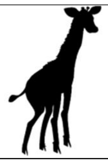	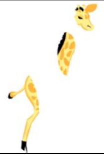
vagn	stroller	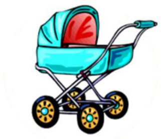	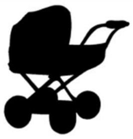	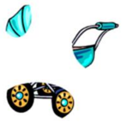
